# Valproic acid affects neurogenesis during early optic tectum development in zebrafish

**DOI:** 10.1242/bio.059567

**Published:** 2023-01-31

**Authors:** Sierra C. Dixon, Bailey J. Calder, Shane M. Lilya, Brandon M. Davies, Annalie Martin, Maggie Peterson, Jason M. Hansen, Arminda Suli

**Affiliations:** Department of Cell Biology and Physiology, Brigham Young University, Provo, UT 84602, USA

**Keywords:** Zebrafish, Valproic Acid, Optic Tectum, Neurogenesis, Axonogenesis, Dendritogenesis

## Abstract

The mammalian superior colliculus and its non-mammalian homolog, the optic tectum (OT), are midbrain structures that integrate multimodal sensory inputs and guide non-voluntary movements in response to prevalent stimuli. Recent studies have implicated this structure as a possible site affected in autism spectrum disorder (ASD). Interestingly, fetal exposure to valproic acid (VPA) has also been associated with an increased risk of ASD in humans and animal models. Therefore, we took the approach of determining the effects of VPA treatment on zebrafish OT development as a first step in identifying the mechanisms that allow its formation. We describe normal OT development during the first 5 days of development and show that in VPA-treated embryos, neuronal specification and neuropil formation was delayed. VPA treatment was most detrimental during the first 3 days of development and did not appear to be linked to oxidative stress. In conclusion, our work provides a foundation for research into mechanisms driving OT development, as well as the relationship between the OT, VPA, and ASD.

This article has an associated First Person interview with one of the co-first authors of the paper.

## Teaser

**Summary:** Optic tectum (OT) is a midbrain structure that is important in integrating multimodal input and guides non-voluntary movements in response to prevalent stimuli. Here we show that valproic acid, associated with autism spectrum disorder, leads to a delay in OT's neurogenesis and neuron specification.

## INTRODUCTION

The superior colliculus (SC) is a mammalian midbrain structure that receives visual, auditory, and somatosensory inputs. It is known for its ability to direct movement of the eyes (Sparks, 1986), ears (Stein and Clamann, 1981), and limbs (Stein and Gaither, 1981) towards salient events in the environment. Engagement of the SC in cross-modal sensory integration leads to an increased ability to detect events (Stein et al., 1989, 1988), a faster reaction time (Amlot et al., 2003; Colonius and Arndt, 2001; Diederich et al., 2003; Frens et al., 1995; Harrington and Peck, 1998; Hughes et al., 1994), and an increased ability to localize targets or events (Hughes et al., 1994; Hairston et al., 2003). Ablation studies have shown that while the visual cortex allows for discrimination of visual inputs, the SC is necessary to initiate movements toward these events (Schneider, 1969). Recently, this subcortical area has been implicated in influencing social behaviors and has been purported to lead to neurodevelopmental disorders such as autism spectrum disorder (ASD) (Burstein and Geva, 2021; Jure, 2018; McFadyen et al., 2020; Solie et al., 2022). Although many studies have shed light on SC function, its microcircuitry and formation continue to be poorly understood.

Work using the genetically-tractable zebrafish model organism has become instrumental in dissecting the microcircuitry of the optic tectum (OT), the non-mammalian homolog of the SC. Similar to the SC, the OT receives sensory inputs and is involved in phototaxis, hunting, and predator avoidance (Nevin et al., 2010, 2008; Wong, 1999; Gahtan et al., 2005; Fero et al., 2011; Lowe et al., 2013; Vanwalleghem et al., 2017). During embryonic and larval development, the OT consists of the stratum periventriculare (SPV), where the neuronal cell bodies reside, and the neuropil, where the neurites extend (Nevin et al., 2010). To better understand OT development, we took a neurotoxicological approach. We reasoned that similar to forward genetic or chemical screens, perturbation of neurodevelopment via a neurotoxin – in our case valproic acid (VPA) – would lead to identification of processes and eventually the genetic and molecular underpinnings required for proper OT development. Valproic acid was chosen due to its use as a model for ASD in zebrafish, characterized by deficits in both social communication and interactions, as well as restricted and repetitive behaviors, interests, and activities. Both genetic and environmental factors have been linked to ASD etiology, and treatment of zebrafish embryos with VPA has been shown to induce similar social interaction deficits (Baronio et al., 2018; Chen et al., 2018; Dwivedi et al., 2019; Zimmermann et al., 2015) and affect ASD-associated genes (Dwivedi et al., 2019).

Valproic acid is a commonly used drug in the treatment of epilepsy, bipolar disorder, and schizophrenia; however, in 1984 it was shown to have adverse effects in the developing fetuses of pregnant mothers taking VPA. These effects were termed ‘fetal valproate syndrome’ (DiLiberti et al., 1984), with ASD being one of the most prominent disorders associated with it. Results from early, smaller, studies showed that VPA monotherapies correlated with much higher rates of ASD (12% rate) compared to unexposed groups (1.9% rate) and were significantly higher than other antiepileptic drugs (both carbamazepine and lamotrigine), which resembled unexposed groups (Bromley et al., 2013). As more information becomes available, including that from larger studies, data shows that VPA exposure not only affects morphological endpoints during development, such as neural tube defects, but is also associated with the prevalence of ASD, confirming the results of the earlier studies. In fact, the risk of developing ASD for individuals born to mothers exposed to VPA during pregnancy is fivefold higher than the general population (Christensen et al., 2013). Mechanisms of VPA-induced developmental toxicity are not fully understood, but potential contributors include VPA-induced oxidative stress and histone deacetylase (HDAC) inhibition.

In our study, we exposed zebrafish embryos to VPA from the onset of gastrulation, 6 h post fertilization (hpf), to 120 hpf and focused specifically on OT development. We show that VPA exposure leads to stalled neurogenesis and perturbation of neuropil formation. Additionally, we identify a critical window for the effect of VPA exposure on the OT. Finally, we demonstrate that these effects do not seem to be caused by oxidative stress, since pretreatment with 3H-1, 2-dithiol-3-thione (D3T), a drug that induces protective stress-response genes, does not ameliorate the effects of VPA.

## RESULTS

### Morphological changes in the OT following VPA exposure reveal developmental delays

**Fig. 1.**
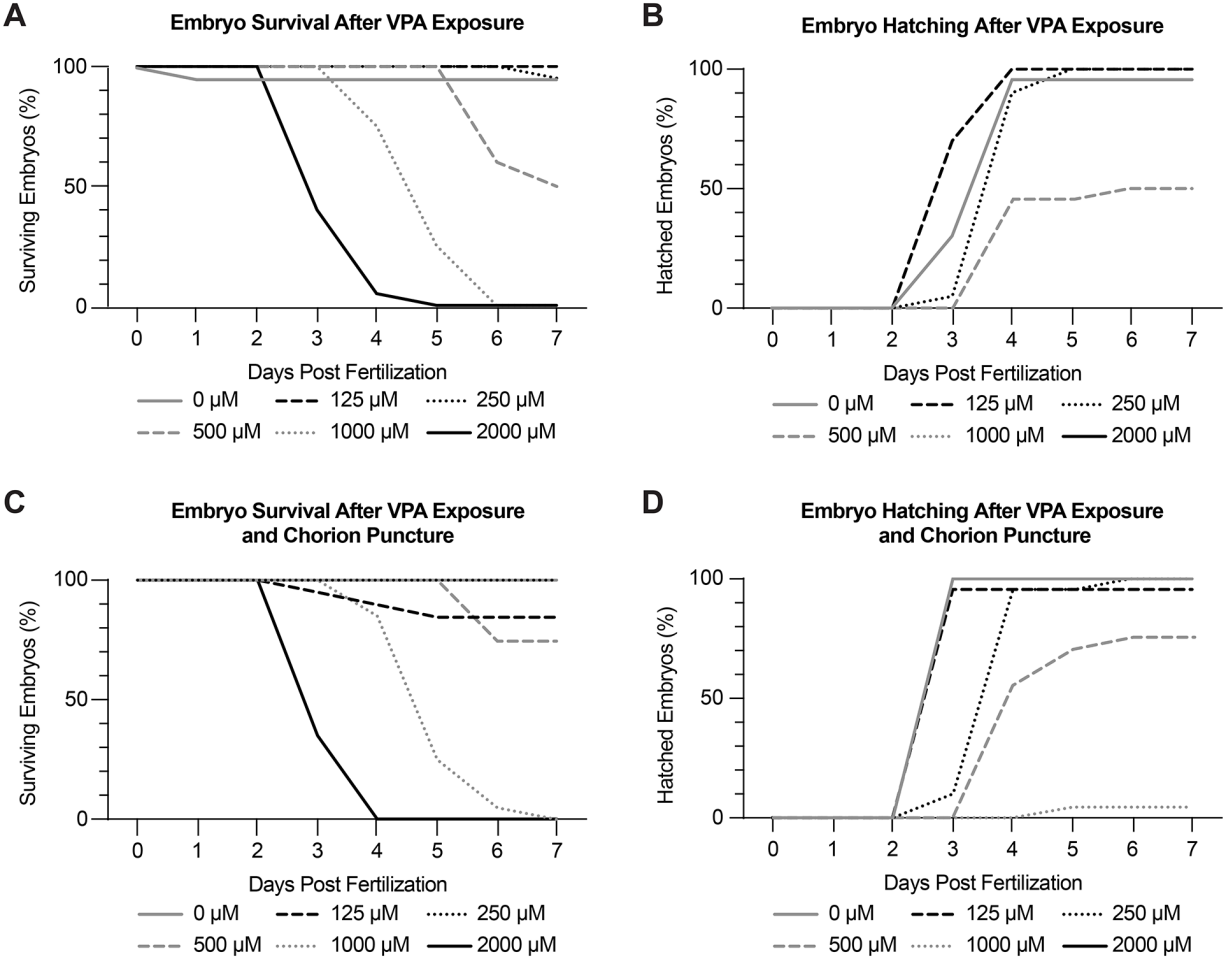
**Dose-response experiments identify 250 μM VPA as the maximum tolerated dose (MTD).** Embryo survival (A) and hatching (B) were recorded daily from 0-7** **dpf using concentrations of VPA ranging from 0-2000 μM. Treated embryos were continuously exposed to VPA from 6-120 hpf. 250 μM was found to be the MTD due to high embryo survival (A) and hatching (B). (C-D) Because zebrafish embryos are encased in a chorion until around 72 hpf, we wondered if VPA was not able to reach the embryos while they were in their chorion. As a result, we repeated the dose-response experiments in embryos where the chorion was punctured. We found no appreciable difference in survival or hatching in embryos with intact or punctured chorions, showing that we could treat the embryos during the early stages without having to remove the chorion. Experiments were repeated twice with 10 embryos per treatment each time.

In order to determine the optimal VPA dose, which would perturb OT development without inducing high mortality rates, dose-response experiments were performed at various concentrations ranging from 0-2000 µM VPA (Fig. 1A,B). Embryos were treated continuously from 6-120 hpf, and the VPA solution was replaced every 24 h. Embryo survival and hatching was recorded daily, with 250 µM identified as the maximum tolerated dose (MTD) due to its high survival and hatching rates. Similar results were found in embryos following chorion puncture (Fig. 1C,D), indicating that the chorion did not prevent the effects of VPA on development. As such, all subsequent experiments were carried out without chorion puncture. In this paper, zebrafish from 0-71 hpf will be termed embryos and from 72-120 hpf will be termed larvae. For any reference that spans the entirety of 0-120 hpf we will use the term embryo.

**Fig. 2.**
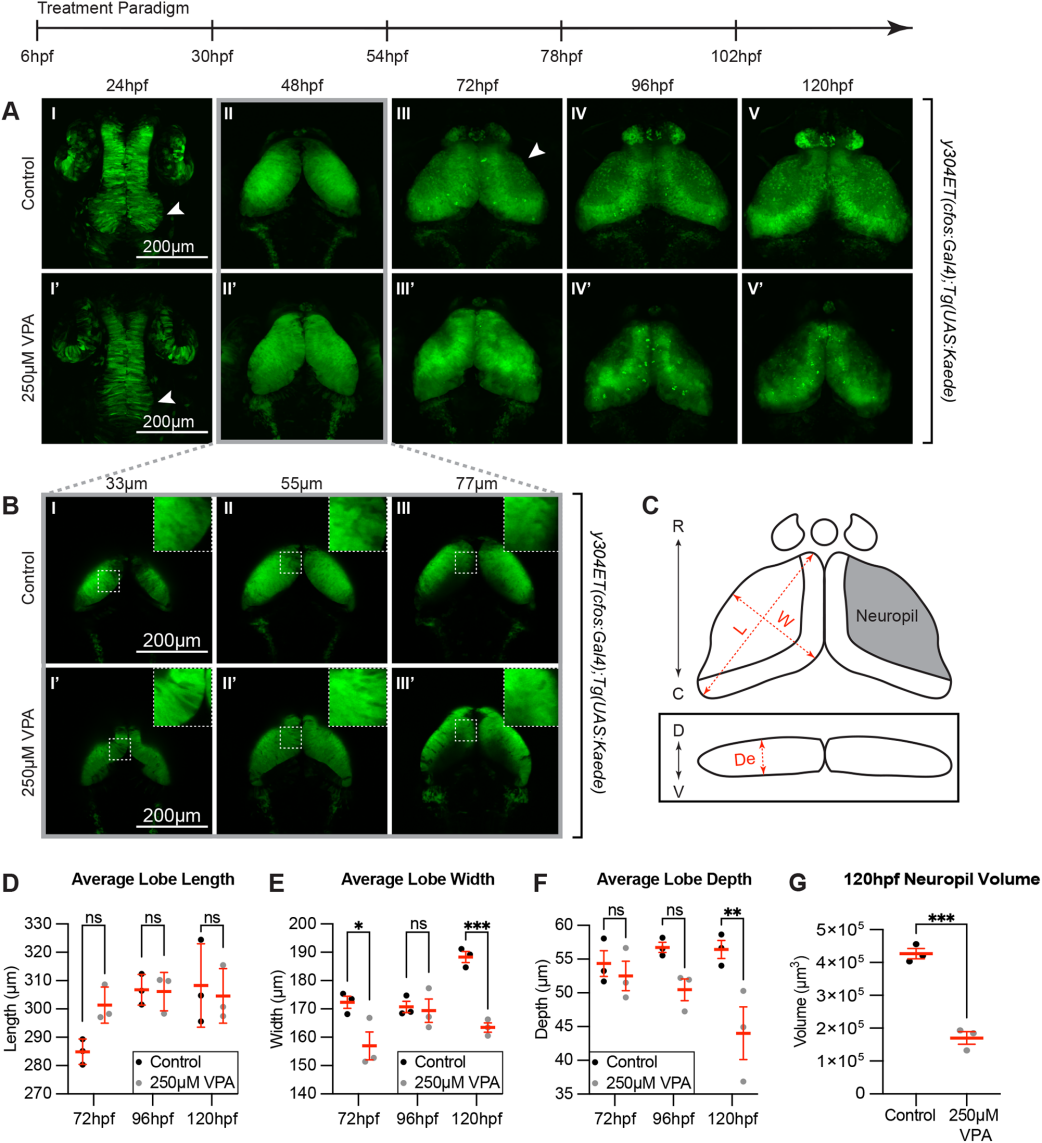
**Continuous VPA treatment causes delayed OT development and decreased neuropil.** (A) Daily images of control (I-V) and 250 µM VPA-treated *y304Et(cfos:Gal4); Tg(UAS:Kaede)* embryos (I′-V′). Treated embryos were continuously exposed to VPA from 6-120 hpf. At 24 hpf, proliferation in the neuroepithelium giving rise to the OT is seen in control (arrow, I) but not in treated embryos (arrow, I′). Beginning at 72 hpf, the OT neuropil, where the neurites extend, becomes noticeable in control (arrow, III) but not in treated larvae (III′). (B) Images of single slices of dorsal (33 µm) to ventral (77 µm) positions at 48 hpf in OT of control (I-III) and VPA-treated embryos (I′-III′). Neurogenesis in treated embryos appears delayed as shown by the presence of columnar neuroepithelial cells dorsally in treated embryos (I′, inset in I′) but not in control embryos where neuroprogenitors and early-born neurons (rounded cells) have taken their place (I, inset in I, and Fig. 3; insets are zoomed in areas from the dashed-line ROIs). (C) Cartoon of OT lobe measurements of length, width, and depth. (D) The lobe length of treated larvae is not significantly different than controls. R: rostral, C: caudal, D: dorsal, V: ventral, L: length, W: width, De: depth. (E) Lobe width of treated embryos is significantly decreased at 72 hpf and 120 hpf, while depth is significantly decreased at 120 hpf (F). (G) Neuropil volume is decreased in VPA-treated larvae at 120 hpf. Although these observations were seen in three different week-long experiments (three embryos per experiment and experimental condition), the quantification was done in three embryos from the same experimental week. Different embryos/larvae were imaged each day and euthanized at the end of the imaging session. Data are shown as mean±s.e.m. A two-way ANOVA with Šidák's multiple comparisons test was performed in D-F, and a two-tailed unpaired t-test was performed in G. At 48 hpf, length, width, and depth are difficult to measure since during this time neuroepithelium is transitioning into neuroblasts. *P<0.05; **P<0.01, ***P< 0.001.

The *y304Et(cfos:Gal4); Tg(UAS:Kaede)* enhancer trap line (Marquart et al., 2015) labels the OT, as well as epiphysis, habenula, heart, and (sparsely) the olfactory bulbs. Therefore, this transgenic line was used to determine the morphological effects of VPA treatment during OT development. *Y304Et(cfos:Gal4); Tg(UAS:Kaede)* embryos were treated continuously with 250 µM VPA from 6-120 hpf (Fig. 2A). VPA was replaced every evening and embryos were imaged the following morning from 24-120 hpf.

**Fig. 3.**
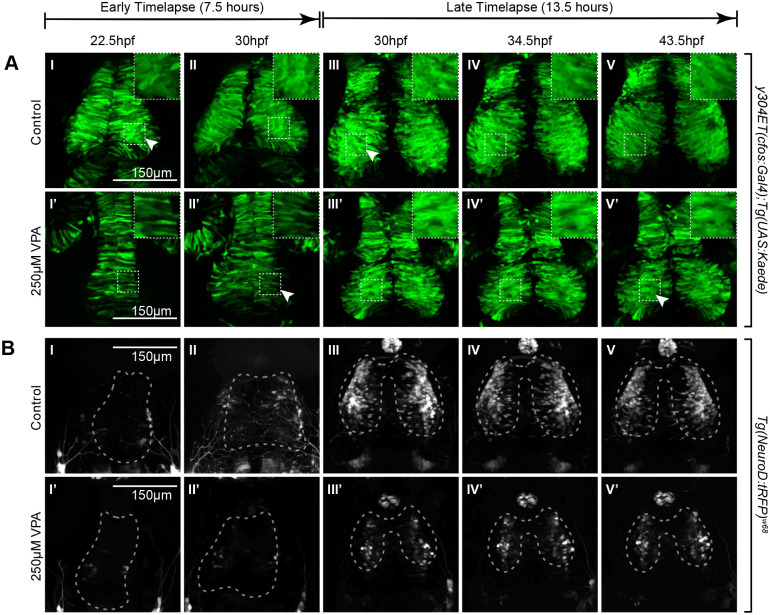
**Timelapse imaging between 22.5-30 hpf and between 30-43.5 hpf shows a delay in neuronal differentiation and specification in VPA-treated embryos.** (A I-II, I′-II′) Timelapse imaging of VPA-treated and control *y304Et(cfos:Gal4); Tg(UAS:Kaede)* embryos at 22.5hpf-30 hpf show that neuroepithelium proliferation (columnar cells) and early neuron generation/differentiation (shorter, rounder cells) is lagging in treated embryos (I′-II′) and is more comparable (arrow, II′) to controls at earlier time points (arrow, I). Insets show a magnified view of the ROIs in dashed lines. (B I-II, I′-II′) At 22.5-30 hpf, no appreciable neuronal specification is seen in either control (I-II) or treated embryos (I′-II′) as determined by lack of fluorescence in the *Tg(NeuroD:tRFP)^w68^* line (dashed outlines indicate OT location). (A III-V, III′-V′) At 30-43.5 hpf, neuroepithelium proliferation and neuron generation/differentiation continues to lag in treated embryos (arrow, V′) when compared to controls (arrow, III). During this time, more neurons become specified in controls (B III-V) when compared to VPA-treated embryos (B III′-V′). Fluorescence in controls condenses into the shape of the OT as the timelapse progresses (B III-V). No increase in fluorescence is apparent for the treated embryos through the timelapse duration. (B III′-V′). For each timelapse, the experiment was repeated twice, and three embryos were timelapse-imaged every time. (A II-III, II′-III′; B II-III, II′-III′) Discrepancies in developmental stages between the movies of the two time periods of the same transgenic line result from delays imposed from time away from a controlled incubation environment as the timelapse progressed.

At 24 hpf, control embryos exhibit a thickening of the neuroepithelium at the posterior part of the neural tube, indicating cell proliferation and the onset of neurogenesis, which is absent in VPA-treated embryos (Fig. 2A I,I′, arrows). By 48 hpf, z-projected images of both control and VPA-treated embryos display similar OT formation (Fig. 2A II,II′). However, individual images taken at the same depth show that dorsally, the OT of VPA-treated embryos exhibit persistent neuroepithelium (columnar cells) undergoing neurogenesis (Fig. 2B I′), while ventrally neuroprogenitor cells and newly born neurons (rounded cells) are present (Fig. 2B III′) (we looked more closely at this in the next experiment). In contrast, control embryos do not show the presence of neuroepithelium at any dorsal to ventral position, indicating that neurogenesis is well underway (Fig. 2B I-III). The developmental delay became even more apparent at 72 hpf when the control larvae show the beginning of neuropil formation (Fig. 2A III, arrow), indicating that neurons are undergoing axonogenesis and dendritogenesis. The neuropil in VPA-treated larvae seems to be absent at 72 hpf and is smaller by 120 hpf (Fig. 2G). For a more quantitative assessment of the phenotype, we measured the length, width, and depth of each OT lobe (Fig. 2C-F). VPA-treated larvae showed decreased lobe width at 72 hpf and 120 hpf, further evidencing a decrease in neuropil volume (Fig. 2E,G), and decreased lobe depth at 120 hpf (Fig. 2F). These observations suggest that decreased or delayed neurogenesis and neuropil formation may be two mechanisms by which VPA alters OT development.

In addition to concentration, previous findings have shown the importance of pH in modulating the effects of VPA and its uptake into the cell (Terbach et al., 2011). In agreement with these studies, we found that the same dose of VPA at a lower pH (pH=6.6) exhibited stronger morphological phenotypes than those at a neutral pH (pH=7.2). In comparison, those at higher pH (pH=7.8) displayed weaker phenotypes (Fig. S1). To keep the phenotype consistent, all subsequent experiments were carried out at a neutral pH (pH=7.2), which was chosen because of its proximity to biological pH and because it is neutral enough to not alter the effects of VPA treatment.

### VPA delays neurogenesis in the developing OT

To better understand the effect of VPA exposure on neurogenesis, we used the *Tg(NeuroD:tRFP)^w68^* transgenic line which drives tRFP via the *NeuroD1* enhancer. *NeuroD1* has been shown to be involved in neuronal differentiation, specification, and migration within the developing mouse cortex (Tutukova et al., 2021), making it a good indicator of neurogenesis. Additionally, single-cell RNA sequencing (scRNA-seq) data previously collected in our lab (Martin et al., 2022) confirmed the presence of *NeuroD1* in the zebrafish OT and showed that its expression is sequestered to specific populations by 7dpf (Fig. S2A). To capture initial alterations in neurogenesis before large divergences in morphology occurred, we ran two consecutive timelapses, which in their entirety covered 22.5-43.5 hpf (Fig. 3). These timepoints were chosen because, as mentioned above, between 24-48 hpf the neuroepithelium, from which the OT is derived, undergoes initial neurogenesis and gives rise to newly born neurons (Fig. 2A I-II, B I-III). Timelapses were run on both *y304Et(cfos:Gal4); Tg(UAS:Kaede)* (Movies 1 and 2) and *Tg(NeuroD:tRFP)^w68^* embryos (Movies 3-5), following the same dosing paradigm used previously, to allow for visualization of cell movement and morphology as well as identify regions undergoing neurogenesis. During the earlier timelapse (22.5-30 hpf) a considerable morphological delay was observed, with the OT of VPA-treated embryos at 30 hpf (Fig. 3A II′) resembling that of control embryos at 22.5 hpf (Fig. 3A I). However, despite these alterations in morphology, no appreciable neurogenesis (tRFP expression) was observed in the region of the OT until 30 hpf in control embryos (Fig. 3B II), and no appreciable neurogenesis was observed at all in VPA-treated embryos during the earlier timelapse (Fig. 3B I′-II′). During the later timelapse (30-43.5 hpf) (Fig. 3A III-V, III′-V′) developmental delay is again evident in the overall OT morphology of VPA-treated embryos when compared to control. Images of 30 hpf control embryos (Fig. 3A III, arrow), where the transition from a columnar shape to the rounder cells characteristic of differentiating neurons has begun, resemble those of 43.5 hpf VPA-treated embryos (Fig. 3A V′, arrow), indicating a delay in this transition. This delay becomes more evident when comparing *Tg(NeuroD:tRFP)^w68^* images (Fig. 3B III-V, III′-V′), which show a substantial decrease in neurogenesis in the region of the OT in VPA-treated embryos compared to control embryos at all imaged time points. Note: Discrepancies in *Tg(NeuroD:tRFP)^w68^* expression at 30 hpf for the second timelapse (Fig. 3A II, II′, B II, II′) compared to the same time point during the first timelapse (Fig. 3A III, III′, B III, III′) are due to delays imposed from time away from a controlled incubation environment as the timelapse progressed.

**Fig. 4.**
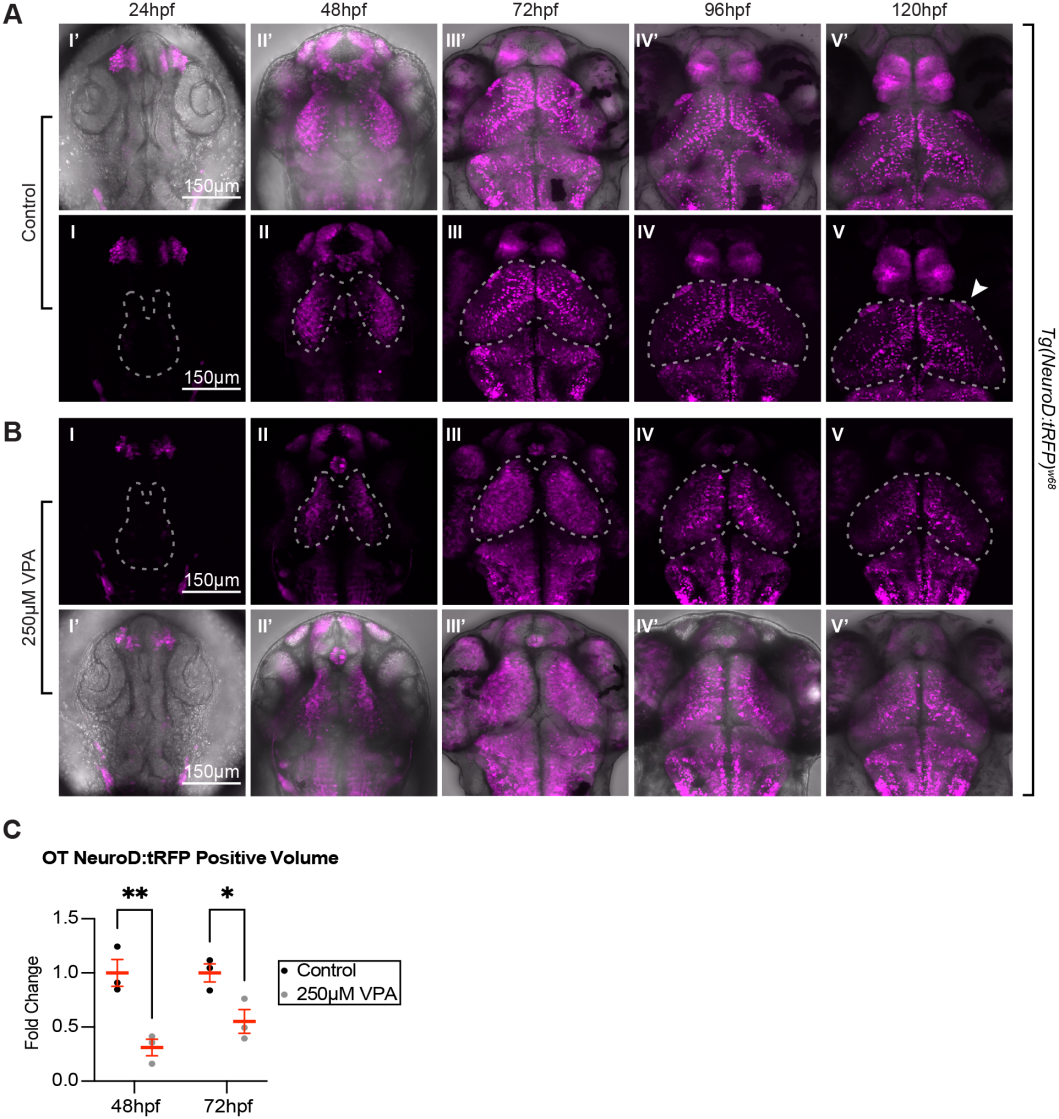
***Tg(NeuroD:tRFP)^w68^* imaging during the first 5 days of development reveals differences in neuronal maturation between control and treated embryos.** Similar to Fig. 2, we noticed no appreciable neuronal specification (as assessed by tRFP expression) in control (A I, I′) or treated embryos (B I, I′) at 24 hpf, and more neuronal specification in control (A II, II′) versus treated embryos (B II, II′) at 48 hpf (dashed outlines indicate OT location). As development progressed from 72-120 hpf, tRFP began to be restricted to subsets of neurons in control larvae (A III-V, III′-V′). Instead, in treated larvae, tRFP expression was detected throughout the OT at 72 hpf (B III), which most likely resembles a time point between 48 and 72 hpf in control embryos (not imaged). This expression becomes more restricted at 96-120 hpf (B IV,V,IV′,V′), suggesting that although neuronal specification lags or slows down in treated embryos, it does eventually occur. Whether the OT neurons in treated embryos are specified to become the same subclasses of neurons as in the control embryos remains to be determined. In addition, treated larvae lack expression in arborization field 7 (A F7) (arrow in A V), a pretectal nucleus shown to be involved in hunting behavior, and scattered neuropil expression seen in controls. Panels I′-V′ in both A and B are overlays of fluorescent images on transmitted confocal projections. (C) Quantification of the volume of tRFP positive neurons at 48 hpf and 72 hpf. Since tRFP is localized in the cytoplasm, it wasn't possible to count tRFP positive neurons. Therefore, we quantified the tRFP positive volume of control and treated embryos, and found that the tRFP positive volume is significantly smaller in treated embryos when compared to controls. Although these observations were seen in three different week-long experiments (three embryos per experiment and experimental condition), the quantification was done on three embryos from the same experimental week. Data are shown as mean±s.e.m. A two-way ANOVA with Šidák's multiple comparisons test was used for statistical analysis. *P<0.05, **P<0.01.

To visualize the long-term effects of VPA on OT neurogenesis, we imaged *Tg(NeuroD:tRFP)^w68^* control and VPA-treated embryos daily from 24-120 hpf (Fig. 4) using the same treatment paradigm as previously discussed. Consistent with the timelapses (Fig. 3), no appreciable neurogenesis occurred in the region of the OT in either control or VPA-treated embryos at 24 hpf (Fig. 4A I, B I). However, differences began to emerge at 48 hpf with VPA-treated embryos (Fig. 4B II) exhibiting decreased *NeuroD1* expression, as quantified by the volume of neurons expressing tRFP (Fig. 4C), when compared to that of control embryos (Fig. 4A II) in the OT. Interestingly, at 72 hpf *NeuroD1* expression appears more widespread in VPA-treated tecti (Fig. 4B III) when compared to that of control larvae, in which *NeuroD1* becomes restricted to specific areas of the OT (Fig. 4A III). This is consistent with the previously described scRNA-seq data (Fig. S2A) suggesting that at 7 dpf, as the OT matures, *NeuroD1* expression is restricted to only a subset of OT cell types, thus indicating that widespread *NeuroD1* expression in the OT of VPA-treated larvae at 72 hpf is likely indicative of a developmental delay. Despite the wider spread of expression throughout OT neuronal subtypes, the volume of neurons expressing tRFP in VPA-treated embryos was still smaller than in control embryos at 72 hpf (Fig. 4C). Restriction of *NeuroD1* expression is eventually observed in VPA-treated larvae at both 96 hpf and 120 hpf; however, VPA-treated larvae lack the scattered neuropil expression and expression in one of the visual receptive areas: arborization field 7 (AF7), (Fig. 4A V, arrow), a pretectal nucleus shown to be involved in hunting behavior (Semmelhack et al., 2014; Antinucci et al., 2019; Baier and Wullimann, 2021), seen in control larvae (Fig. 4A IV-V, IV′-V′). While restriction of *NeuroD1* expression, indicating neural specification, does eventually occur in VPA-treated larvae (Fig. 4A IV-V), it remains to be determined if OT neurons are specifying into the proper neuronal subtypes.

### VPA affects the emergence of different neuronal subtypes in the developing OT

**Fig. 5.**
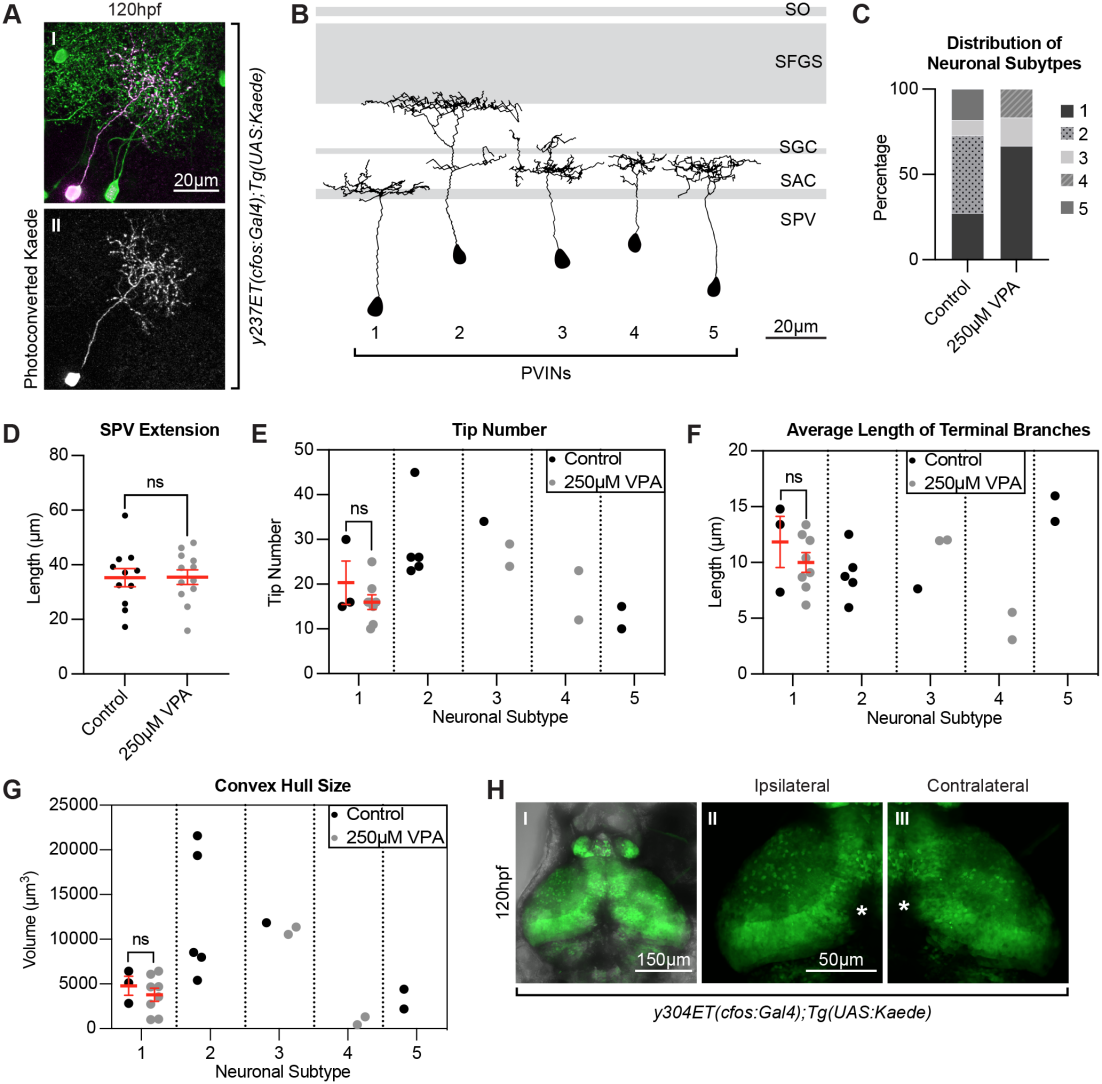
**Photoconversions of neurons in the OT of VPA-treated embryos reveal shortened and less complex neuronal projections.** (A) Photoconversion of Kaede (II) from green to red (pseudo-colored magenta) (I) was carried out in single neurons in 5 dpf larvae in the *y237Et(cfos:Gal4); Tg(UAS:Kaede)* line, which labels only a few OT neurons. Eleven neurons from control larvae and twelve neurons from 250μM VPA-treated larvae were photoconverted and traced using the SNT macro, Fiji (ImageJ). (B) The presence of five different periventricular interneuron (PVIN) subtypes which extend projections in different layers of the neuropil were detected. Panel based on (Nevin et al., 2010) with OT retinoreceptive laminae shown in gray. SPV: stratum periventriculare; SAC: stratum album centrale; SGC: stratum griseum centrale; SFGS: stratum fibrosum et griseum superficiale; SO: stratum opticum. (C) Most of the photoconverted neurons in control larvae were of subtype 2 [which extend their projections to the more distal laminae SFGS (*n*=5)] and subtype 1 (*n*=3), fewer of subtype 5 (*n*=2) and subtype 3 (*n*=1), with no subtype 4 neurons. In the treated larvae, most of the neurons were of subtype 1 (*n*=8) and few of subtype 3 (*n*=2) and subtype 4 (*n*=2), with no subtype 2 or subtype 5 neurons. (D) The distance of the photoconverted PVIN cell bodies to the start of the neuropil, on the other hand, was not different between control and treated larvae. All neuronal subtypes were combined for this analysis (control *n*=11) (250 µM VPA, *n*=12). Data are shown as mean±s.e.m. A two-tailed unpaired *t*-test was used to determine significance. *P<0.05, **P<0.01, ***P<0.001. Using SNT macro, Fiji (ImageJ), on the traced neurons for each neuronal subtype in control and treated conditions, we calculated the tip number (E), average length of terminal branches (F), and convex hull size (G), which indicates the total volume of the arborization field. Since we did not have enough neurons representing each subtype in control and treated conditions, we only compared these parameters in subtype 1 neurons and found them to be not statistically different from one another. (Each dot represents one neuron). Data shown as mean±s.e.m. A two-tailed unpaired *t*-test with Welch's correction was used to determine significance ***P<0.001. (H I) Unilateral enucleations (eye removals) (left), show that both ipsilateral (H II) and contralateral (H III) OT neuropils are identical to each other, indicating that the status of retinotectal axons is not integral to the initial development of OT neuron projections. This suggests that VPA effects on the OT are not due to possible VPA effects on retinotectal projections from the retina, as seen in other studies (Cowden et al., 2012). (H II and H III) zoomed in views of H I. * in H II and H III indicate pigment spots concealing the structures below.

To start exploring the mechanism by which VPA prevents neuropil formation, we next used photoconversion of Kaede from green to red to visualize neurons and their projections in control and VPA-treated larvae (Fig. 5A). Our scRNA-seq shows the presence of a minimum of 25 different subgroups of neurons by their expression profiles in the *y304Et(cfos:Gal4); Tg(UAS:Kaede)* line (Martin et al., 2022). This makes it difficult to label by photoconversion and visualize the same neuronal subtypes in the *y304Et(cfos:Gal4); Tg(UAS:Kaede)*. As a result, we performed the photoconversion experiments in a different transgenic line, *y237Et(cfos:Gal4); Tg(UAS:Kaede)*, which shows sparser Kaede labeling in the OT. Control and VPA-treated *y237Et(cfos:Gal4); Tg(UAS:Kaede)* embryos underwent the previously discussed treatment paradigm and were incubated in the dark to prevent photoconversion. Kaede was photoconverted (Fig. 5A) in 60 individual neurons in control larvae and 37 individual neurons in VPA-treated larvae at 5 dpf. Out of these, only 11 control neurons and 12 VPA-treated neurons had optimal red Kaede expression and presence of neurite projections in distinct laminar layers. After image deconvolution (Huygens), Fiji's (ImageJ) SNT macro was used to trace all the neurites extending from each cell body for the 23 total neurons in control and VPA-treated conditions. The presence of five different periventricular interneuron (PVIN) subtypes that extend projections in different layers of the neuropil were detected (Fig. 5B, Movies 7-11). Almost half of the photoconverted neurons in control larvae were of subtype 2 (*n*=5), which extend their projections to the most distal retinoreceptive lamina, SFGS (Fig. 5C). The rest were of subtype 1 (*n*=3), subtype 5 (*n*=2), and subtype 3 (*n*=1), which innervated the more proximal laminae: SAC and SGC. In the VPA-treated larvae, all of the neurons were of subtype 1 (*n*=8), subtype 3 (*n*=2), and subtype 4 (*n*=2), which extend their projections in the more proximal retinoreceptive areas: SAC and SGC. No photoconverted neurons were subtype 2 neurons in the VPA-treated embryos. Although the numbers of the photoconverted neurons are not extensive, the failure to detect any of the (distally-extending-neurite) subtype 2 neurons in VPA-treated *y237Et(cfos:Gal4); Tg(UAS:Kaede)* larvae could explain the reduced neuropil in VPA-treated larvae when compared to controls in the *y304Et(cfos:Gal4); Tg(UAS:Kaede)* line (Fig. 2G). Presently, we cannot determine if the possible lack of subtype 2 neurons in the VPA-treated larvae is a result of failure or delay in specification of these neurons. Interestingly, the distance of the photoconverted PVIN cell bodies from the start of the neuropil, was not different between control and treated larvae (Fig. 5D), showing that the neuronal cell bodies we photoconverted are found at similar positions within the periventricular zone. Finally, when comparing parameters such as the terminal branch tip number (E), average length of terminal branches (F), and convex hull size (G) (which indicates the total volume of the arborization field) in subtype 1 neurons (the only ones found in sufficient numbers in control and VPA-treated conditions for statistical purposes), we found no statistical differences between conditions. Without additional numbers of traced neurons, it is difficult to determine if VPA affects the complexity of subtype 3, 4, and 5 neurons, but the current data strongly suggests that subtype 2 neurons are either absent or developmentally delayed.

The mechanisms underlying OT development are not fully understood, and likely rely on input from various sensory systems. Previous studies have shown that VPA exposure can lead to almost complete loss of retinotectal projections in a dose dependent manner (Cowden et al., 2012). To determine if the loss of retinotectal extensions from the eye due to VPA exposure is responsible for the lack of neuropil seen in the OT of VPA-treated embryos, we imaged *y304Et(cfos:Gal4); Tg(UAS:Kaede)* embryos at 120 hpf following unilateral enucleation (eye removal) at 32 hpf (Fig. 5H). Comparison of ipsilateral (Fig. 5H II) and contralateral (Fig. 5H III) tectal lobes showed no apparent difference in neuropil formation. These results indicate that VPA's effects on neuropil and neuronal development/specification in the OT are independent of VPA's effect on retinotectal projections.

### VPA effects do not extend to all neurons in the embryo

**Fig. 6.**
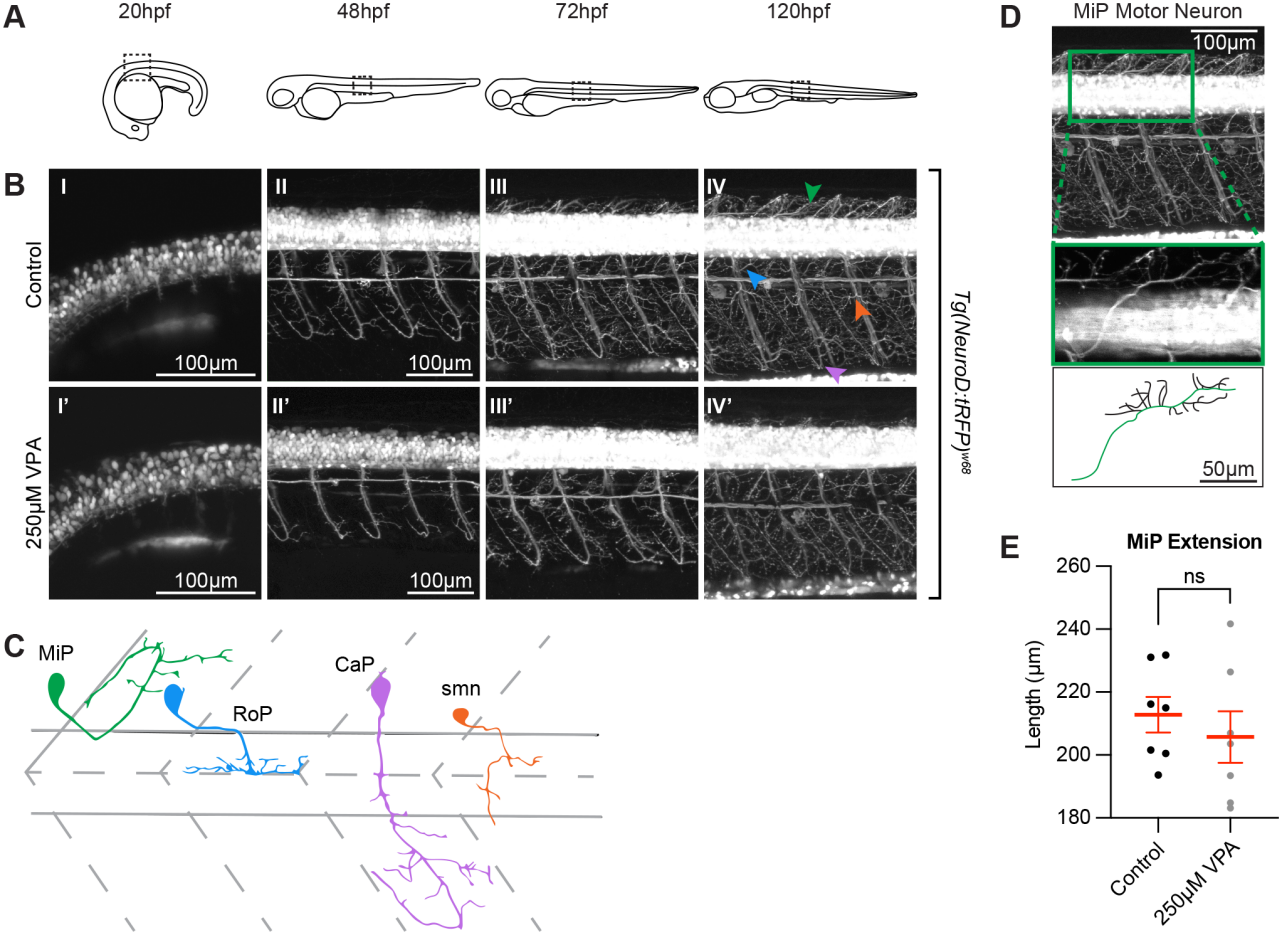
**VPA effects do not extend to all neurons in the embryo.** (A) Schematic of the imaging location at the different time points in embryos and larvae. Images of the spinal cord neurons in the *Tg(NeuroD:tRFP)^w68^* line indicate that neurogenesis (B) is occurring at the same time (∼20 hpf) in control (I) and treated embryos (I′). Moreover, despite being somewhat delayed, axonogenesis and dendritogenesis in treated embryos (II′-IV′) does not look significantly different when compared to controls (II-IV) from 48-120 hpf. (C) Cartoon of MiP, RoP, CaP, and smn motor neurons based on (Myers et al., 1986). Initially we thought that the dorsal projections of MiP motor neurons in the treated larvae were affected. However, when we used Fiji's (ImageJ) SNT macro, to trace the neurons in three dimensions (D), and measure the length of the primary projection (green trace in the cartoon), we found that the differences between control and VPA-treated embryos were not statistically different from one another (E). Two to three MiPs were traced in each embryo and three embryos per condition were analyzed. (Each dot represents one MiP). Data shown as mean±s.e.m. A two-tailed unpaired *t*-test was used to determine significance. (*) in IV′ indicate pigment spots concealing the structures below. Due to the high fluorescence level in the neuronal cell bodies, gamma has been adjusted (0.60) uniformly on all images in panel B to allow for visualization of neuronal projections.

After observing the effects of VPA on differentiation as well as on neuropil formation and complexity within the OT, we next investigated if these effects were regionally localized to the OT or more generalized. To determine the effect of VPA in other regions, we imaged the spine of *Tg(NeuroD:tRFP)^w68^* VPA-treated and control embryos (Fig. 6) using the treatment paradigm previously discussed. Starting at 20 hpf and continuing through 120 hpf VPA-treated embryos showed a slight delay in development compared to their control counterparts, characterized by a seeming decrease in complexity and extension of RoP, CaP and smn motor neurons (Myers et al., 1986) (Fig. 6B II-III, II′- III′), but these delays were overcome by 120 hpf (Fig. 6B IV, IV′). Because it is too difficult to separate the projections of these neurons from one another due to their overlapping neurite projections (Fig. 6C), we did not quantify this phenotype. Looking at the images, we initially thought that MiP motor neurons were shortened in the VPA-treated embryos. Since dorsal projections of MiPs were easier to identify (Fig. 6D), we traced the primary projection of MiP motor neurons (Fig. 6D, green neurite) at 120 hpf in three dimensions using the SNT macro [Fiji (ImageJ)] but found no statistical difference between the MiPs in the two conditions (Fig. 6E). These results show that the effects of VPA treatment during OT development are more severe than those in other neural structures, or at least in spinal cord neurons.

### The critical window of OT susceptibility to VPA-treatment includes timepoints prior to 72 hpf

**Fig. 7.**
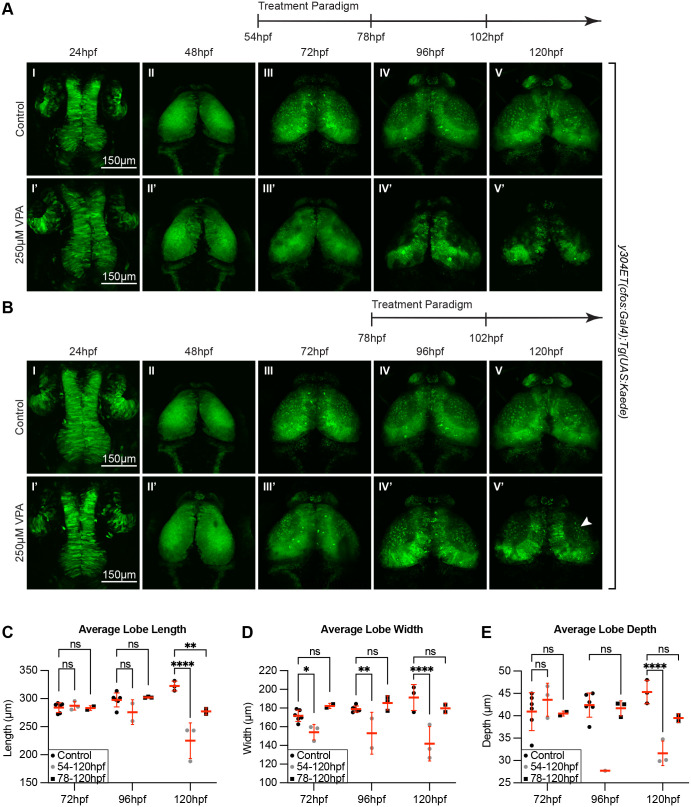
**The critical window of OT susceptibility to VPA-treatment includes timepoints prior to 72 hpf.** (A) Treatment of *y304Et(cfos:Gal4); Tg(UAS:Kaede)* embryos from 54 to 120 hpf still resulted in neuropil development defects in the treated embryos (I′-V′) compared to controls (I-V). Therefore, the critical window of susceptibility must extend beyond 54 hpf. (B) A subsequent trial in which VPA treatment was initiated at 78 hpf resulted in a significantly reduced phenotype in treated larvae (IV′-V′). Three embryos per experimental condition were imaged and quantified. (C) Average lobe length at 120 hpf was significantly decreased for both treatment groups. (D) Average lobe width is consistently decreased for the 54-120 hpf treatment group across all days measured. (E) The average lobe depth is significantly decreased for the 54-120 hpf treatment group compared to controls when measured at 96 and 120 hpf. The 78-120 hpf treatment group shows no significant difference with controls. Therefore, the critical window of susceptibility is determined to be time points prior to 72 hpf. At 48 hpf, length, width and depth are difficult to measure since during this time neuroepithelium is transitioning into neuroblasts. A two-way ANOVA with Šidák's multiple comparisons test was performed. *P<0.05, **P<0.01, ***P<0.001, ****P<0.0001. Data are shown as mean±s.e.m.

We next aimed to determine the critical window during which the OT is highly affected by VPA treatment. Since *NeuroD1* expression began to sequester to specific subpopulations at 72 hpf (Fig. 4A III), indicating completion of initial neurogenesis, and was fully sequestered by 96 hpf (Fig. 4A IV), we identified timepoints prior to 54 hpf or prior to 78 hpf as possibilities for the OT critical window of susceptibility. To test this theory, embryos were dosed following the same paradigm used previously, except that initial VPA treatment was delayed until 54 hpf and 78 hpf respectively. Embryos dosed starting at 54 hpf (Fig. 7A I′-V′) exhibited the overall morphological phenotypes previously observed in full-week treatments (Fig. 2), with almost complete loss of the neuropil at 120 hpf (Fig. 7A V′) and a significant decrease in average lobe length, width, and depth (Fig. 7C-E). When this experiment was repeated with treatment starting at 78 hpf (Fig. 7B) the VPA-induced phenotype was greatly reduced, with no significant differences in average lobe width and depth at 120 hpf (Fig. 7C-E), the presence of some superficial interneurons (SINs) (Fig. 7B V′, arrow), and segregation of the stratum periventriculare (SPV) from the neuropil, all of which were not observed in embryos exposed to full-week treatments.

**Fig. 8.**
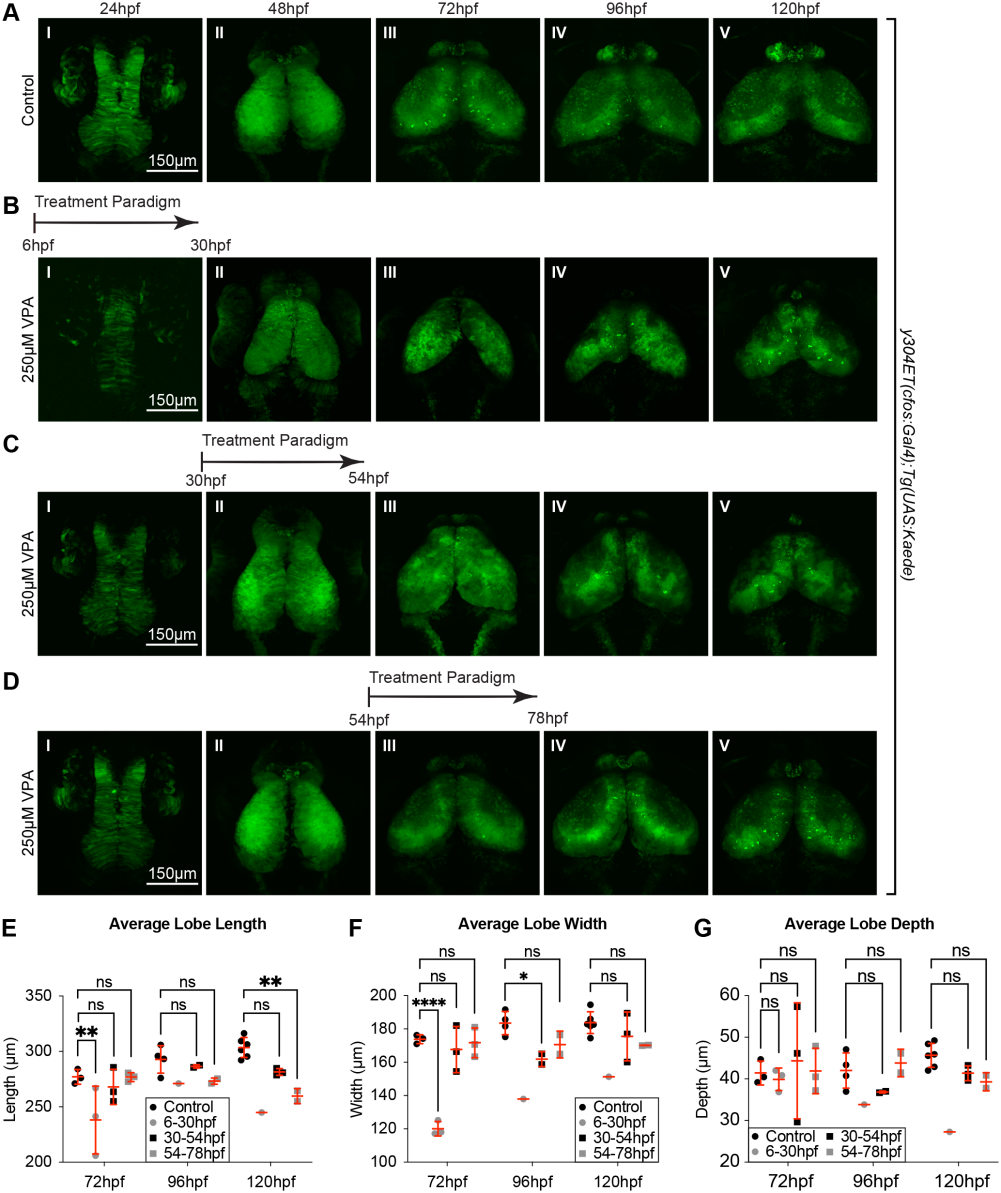
**Single 24 h VPA treatment prior to 78 hpf still result in developmentally delayed OTs.** Short, 24 h VPA treatments were administered during three different time-windows followed by replacement of the solution with EM. Daily imaging of control (A) and 250 µM VPA-treated *y304Et(cfos:Gal4); Tg(UAS:Kaede)* embryos show that one 24 h VPA-treatment at 6-30 hpf (B) or 30-54 hpf (C) still leads to a smaller OT and neuropil (B. II-V, C. III-V, E,F,G) and that the development, despite the shorter treatment time, did not recuperate. (D) When the VPA-treatment was applied between 54-78 hpf, OT development was affected less than in previous treatments (B,C). (E, F,G) Three embryos per experimental condition were imaged and quantified (except for the 6-30 hpf group, where the image from only one embryo prevented analysis at 96 hpf and 120 hpf). At 48 hpf, length, width, and depth are difficult to measure since during this time neuroepithelium is transitioning into neuroblasts. A two-way ANOVA with Šidák's multiple comparisons test was performed in E-G *P<0.05, **P<0.01, ***P<0.001, ****P<0.0001. Data are shown as mean±s.e.m.

To further validate the existence of a critical window, and to determine at what time point the embryos could recuperate from the effects of VPA, we imaged *y304Et(cfos:Gal4); Tg(UAS:Kaede)* embryos before and after single-day VPA treatments starting at 6 hpf, 30 hpf, and 54 hpf. Embryos exposed to single-day VPA treatment at all tested timepoints exhibited developmental delays with embryos treated starting at 6 hpf (Fig. 8B) showing deficits in OT length and width at 72 hpf (Fig. 8E-F). Embryos treated at 30 hpf (Fig. 8C) showed decreased lobe width at 96 hpf which was recovered by 120 hpf (Fig. 8F). Embryos treated at 54 hpf exhibited decreased lobe length at 120 hpf (Fig. 8E). Finally, all embryos treated prior to 78 hpf exhibited decreased neuropil structure compared to controls (8A-D V). These findings suggest that short exposure prior to 78 hpf delays OT development and, when taken together with previous experiments (Fig. 7), indicate a critical window of susceptibility prior to 78 hpf during which VPA exposure delays OT development.

### Upregulation of the Nrf2 antioxidant response system and increased GSH_Tot_ fails to protect against the effects of VPA on OT development

**Fig. 9.**
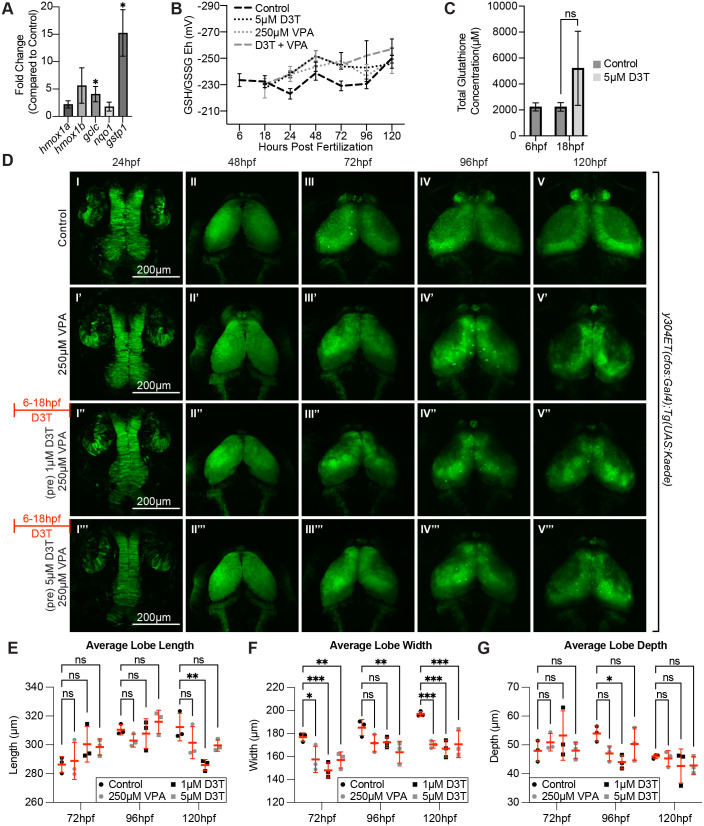
**Induction of genes downstream of the antioxidant-response transcription factor, nrf2, do not protect against VPA effects in the OT.** (A) Treatment of embryos from 6-18 hpf with 5 µM D3T induces expression of some genes (*Gclc* and *Gstp1* that play a direct role in reduced glutathione (GSH) synthesis and usage) downstream of *nrf2* transcription factor gene in total embryos. (B) However, pretreatment for 12 h with 5 µM D3T before incubation with 250 µM VPA did not significantly change the redox potential when compared to VPA only treated embryos at any of the tested time points (C). Data points (A-C) represent *n*=3 pools of 30 embryos. *P<0.05. Data are shown as mean±s.e.m. (D) Pretreatment for 12 h with 1μM (I″-V″) or 5 μM (I′′′-V′′′) D3T before incubation with 250 μM VPA provides no protection in the OT compared to embryos treated with VPA alone (I′-V′) despite upregulation of *nrf2* target genes. (E) Average lobe length is significantly decreased for the 1 µM D3T treatment group, compared to controls, when measured at 120 hpf. (F) For the average lobe width, all treatment groups were significantly decreased compared to controls when measured at 72 hpf and 120 hpf. When measured at 96 hpf the 5 µM D3T treatment groups was significantly decreased compared to controls. (G) Average lobe depth was significantly decreased compared to the controls for the 1 µM D3T treatment group when measured at 96 hpf. At 48 hpf, length, width and depth are difficult to measure since during this time neuroepithelium is transitioning into neuroblasts). In E-G a two-way ANOVA with Šidák's multiple comparisons test was used for statistical analysis *P<0.05, **P<0.01, ***P<0.001. Data are shown as mean±s.e.m.

Previous research done in mouse embryonic teratoma cells identified oxidative stress as a possible mechanism for the negative effects of VPA exposure and demonstrated that upregulation of the nuclear factor-erythroid factor 2-related factor 2 (nrf2) antioxidant response system can ameliorate the negative effects of VPA on neurogenesis *in vitro* (Piorczynski et al., 2022). To investigate the interplay between the nrf2 antioxidant response and VPA in the OT, we administered the nrf2-inducer 3H-1, 2-dithiol-3-thione (D3T) prior to VPA treatment to upregulate expression of specific redox-related genes throughout the embryo. The genes of interest included: *hmox1a, hmox1b, gclc, nqo1, and gstp1*. To verify that D3T was inducing increased expression of these genes, embryos were exposed to D3T for 12 h (6 hpf-18 hpf). Following 12 h of D3T treatment, embryos were collected and prepared for RT-qPCR analysis. When compared to control embryos, *gclc* and *gstp1* showed significantly increased expression (Fig. 9A). Both *gclc* and *gstp1* play a direct role in reduced glutathione (GSH) synthesis and usage, an important molecule in the antioxidant response. Other genes were not significantly different.

To measure redox states after VPA exposure, embryos were treated with 250 µM VPA beginning at 18 hpf and media was replaced every 24 h until 120 hpf. Pools of 30 embryos were collected at 24 hpf, and each following day up to 120 hpf, to be derivatized for HPLC analysis. Expecting an oxidizing shift following VPA treatment compared to control embryos, we also treated embryos with the nrf2 inducer D3T at 6 hpf, prior to VPA treatment. At 18 hpf, D3T was removed and 250 µM VPA was added to some of the embryos following the dosing and collection scheme mentioned above. Interestingly, there was no significant difference in the measured redox potentials between control embryos and embryos treated with D3T, VPA, or D3T+VPA from 6 hpf-120 hpf (Fig. 9B). Additionally, D3T pretreatment did not lead to an increase in total GSH concentration (Fig. 9C, and Fig. S3). To determine the effect of D3T pretreatment on OT phenotype following VPA exposure, *y304Et(cfos:Gal4); Tg(UAS:Kaede)* control, VPA, 1 µM D3T+VPA, and 5 µM D3T+VPA embryos were imaged every 24 h from 24-120 hpf (Fig. 9D) following the dosing paradigm just discussed. At 120 hpf, no improvement was seen between embryos treated with VPA only (Fig. 9D V′) and those pre-treated with either 1 µM (Fig. 9D V″) or 5 µM D3T (Fig. 9D V′′′). Additionally, quantification of tectal parameters showed no rescue following D3T treatment (Fig. 9E-G). Interestingly, pre-treatment with D3T appeared to amplify the negative effects of VPA at 96 hpf and 120 hpf with embryos exhibiting a smaller neuropil than VPA-treated embryos (Fig. 9D IV′-V′, IV″-V″). Furthermore, embryos treated with both 1 µM D3T and 250 µM VPA showed a significant decrease in lobe length not seen in VPA-treated embryos (Fig. 9E). This is in concert with additional experiments showing that prolonged exposure to high concentrations of D3T can impair OT development (Fig. S4). Additionally, timelapse imaging during the initial OT critical period also revealed no rescue in D3T pretreated embryos (Movie 6). These results suggest that D3T pretreatments are not sufficient to ameliorate VPA-induced delays in OT development.

## CONCLUSIONS

Timelapse movies of *y304Et(cfos:Gal4); Tg(UAS:Kaede)* embryos (Movie 1) showed that starting around 22 hpf the neuroepithelium, from which the OT will be derived, undergoes extensive proliferation resulting in neurons that begin to be specified soon after, around 28-30 hpf (Movies 3-5). Embryos treated with VPA show delayed neurogenesis indicated by the delay in *NeuroD1* expression (delay in the appearance of tRFP driven by the *NeuroD1* promoter). From our current experiments, it is difficult to tell if the rate of proliferation or the actual neuronal specification is delayed, but it would be interesting in the future to follow up this observation with timelapse-imaging of embryos that have a nuclear reporter or have been given a BrdU pulse. Previous studies have shown varying results as to whether VPA inhibits or promotes proliferation (Dozawa et al., 2014; Go et al., 2012; Lee et al., 2013, 2019), suggesting that dose and treatment length may alter outcomes. One study (Aluru et al., 2013) of zebrafish embryos treated with VPA identified upregulation of several miRNAs, including those predicted to regulate cell cycle genes. Among those cell cycle regulating genes, *cdkn1a, tp53* are broadly expressed throughout the different neuronal subclasses at 7 dpf in our scRNA-seq data (Martin et al., 2022), while *wee1*, *cdk2* and *chek1* are specifically expressed in one developing neuronal population (Martin et al., 2022) (Fig. S2B). It will be worthwhile in the future to determine if and how these genes regulate OT proliferation during development.

As mentioned above, we found that in control embryos neuronal specification of the OT begins around 28-30 hpf (Movies 3-5). At 72 hpf the tRFP reporter expression is expanded and then restricted at days 4 and 5 to a subset of OT populations. Instead, tRFP expression onset in VPA-treated embryos is delayed from 28-30 hpf to 72 hpf, and then progresses, although slower than controls, in days 4 and 5. This delay in neuronal specification is consistent with findings from neuronal cultures treated with VPA (Piorczynski et al., 2022). It is important to keep in mind that although the delay in neuronal specification seams to become resolved, it is unclear if all the neuronal subclasses are subsequently generated, and if the number for each subclass remains the same. In our single-neuron photoconversion and tracing experiments, in VPA-treated embryos at 5dpf, we found no PVINs that extend their neurites in the most distal lamina SGFS, and which we called subtype 2. Although the numbers of the photoconverted neurons are not extensive, it is intriguing to think that the development of these PVIN subtype 2 neurons might be delayed or absent, which could explain the decreased extension of neuropil found in the VPA-treated larvae. Jacob and colleagues (Jacob et al., 2014) showed that VPA treatment of zebrafish embryos resulted in downregulation of the proneural gene *ascl1b* (associated with Notch signaling) via the blockade of HDAC1 inhibitor, which is required for its expression. Could these same genes play a role in OT neurogenesis? Our scRNA-seq data shows that *HDAC1* and *HDAC4* are indeed present in 7 dpf OT neurons. Although *ascl1b* is not highly expressed in any of the OT neuronal populations at this stage, *ascl1a* and *Her* genes upstream of *ascl1* in the Notch pathway are present in the developing populations (Fig. S2C-D). This suggests that HDAC1, HDAC4 and the Notch pathway might play a role in OT neuronal specification.

The decrease in neuropil extension, does not seem to depend on the potential lack of retinotectal projections from the retinal ganglion cells, which were previously shown to be affected by VPA (Cowden et al., 2012). Early removal of one of the eyes does not appear to significantly affect the contralateral tectal neuropil at 5 dpf, indicating that the decreased extension of the neuropil we observe is most likely due to VPA's effect on OT neurons. Therefore, a more plausible explanation for this phenotype is that VPA affects the development of neuronal subtypes, such as the ones we called subtype 2 PVIN neurons. Although VPA treatment affects OT neuron development, the development of the spinal cord neurons (Myers et al., 1986) are slightly delayed, but able to catch up. This suggests that the development of OT and spinal cord neurons might be overseen by genes that have non-overlapping functions.

Finally, we wanted to determine if the effects of VPA on OT development were due to oxidative stress. The transcription factor nrf2 plays a key role in the expression of many detoxifying and antioxidant enzymes as well as GSH biosynthesis enzymes to resist oxidative stress during differentiation (Venugopal and Jaiswal, 1998; Itoh et al., 1997). Under normal conditions, nrf2 goes through a controlled degradation process dependent on Kelch like ECH-associated protein 1 (Keap1), a nrf2-specific adaptor protein for the Cul3 ubiquitin ligase complex (Kobayashi et al., 2004; Itoh et al., 2003, 1999). Past studies have shown that various chemicals, including D3T, are nrf2 activators through their interactions with KEAP1 and cause the dissociation of KEAP1/nrf2 complex to allow nrf2 to subsequently accumulate in the nucleus and regulate gene expression (Piorczynski et al., 2022; Itoh et al., 2003; Li et al., 2016; Kobayashi et al., 2009). This response system was hypothesized to be a protective mechanism against VPA exposure during zebrafish OT development. However, in the current study, we show that D3T does not have a protective effect in the OT. Although the GSH_Tot_ was increased and various *nrf2*-regulated genes showed a significant increase in expression after D3T pre-treatment, this heightened concentration of GSH and GSSG did not prevent malformation in the OT following VPA exposure. The RT-qPCR and HPLC data represent whole embryos and not solely the OT, so it is unclear what direct effect D3T pretreatments may have on the OT. The results may be different if the redox potential and gene expression exclusively of the OT were measured, which are likely the focus of future experimentation. It would be expected from the data shown in this study that both the redox potential and gene expression remain the same when compared to VPA-treated embryos.

Alternatively, it could be that the expression of nrf2 itself is not high enough early in development when we exposed embryos to VPA. One study showed no significant upregulation of nrf2-regulated genes (namely *gstp1*) nor nrf2 activation at 8-10 hpf after exposure to a wide range of chemicals without the addition of exogenous nrf2 (Kobayashi et al., 2009). The exposure to an oxidant this early in development may come prior to any antioxidant response pathway being robust enough to ensure a functional, beneficial, response and may cause malformation, disruption of protein function, and ROS-mediated damage with limited protection available. Since VPA-induced effects could not be resolved by pretreatment with D3T, there is a strong possibility that the observed phenotypes are not due to oxidative stress, but rather to a possible inhibitory effect on HDAC and an aberrant effect on gene expression profiles. As previously mentioned, HDAC1 and HDAC4 are present in OT at 7 dpf and their specific status in the OT following VPA treatment should be followed up in the future.

In conclusion, our study used VPA to perturb development of the OT for the purpose of eventually understanding the mechanisms underlying proper development of the OT. Our results indicate that VPA treatment delays neurogenesis and potentially impacts the generation of specific subclasses of neurons. Additionally, our findings identify for the first time a critical period for neurogenesis in the OT. Finally, this work provides a foundation for future research into mechanisms driving OT development as well as the relationship between the OT, VPA, and ASD.

## MATERIALS AND METHODS

### Zebrafish lines and husbandry

Embryos and larvae were raised on a 14 h:10 h light:dark cycle. Both sexes were used for these studies due to an inability to tell sex at these developmental stages. All embryos were also homozygous for a mutation in the tyrosinase gene (*tyr-/tyr-*), generated by CRISPR-Cas9 in our lab, which lack pigment formation, to allow for imaging of the optic tectum without obstruction from pigment.

The *y304Et(cfos:Gal4); Tg(UAS:Kaede)* and *y237Et(cfos:Gal4); Tg(UAS:Kaede)* enhancer trap lines (Marquart et al., 2015) were generously provided by the lab of Harold Burgess at NIH.

The *Tg(NeuroD:tRFP)^w68^* line was generated using the Multisite Gateway system (Kwan et al., 2007; Villefranc et al., 2007) to make the NeuroD:tRFP construct from 1) pME NeuroD (a gift from Teresa Nicolson, Department of Otolaryngology/Head & Neck Surgery at Standford; USA, containing the 5 kb region of *Neurod1* 5′promoter Obholzer et al., 2008 2) pME tagRFP (tRFP) (a gift from Chi-Bin Chien) and 3) p3E polyA (Multisite Gateway system). The construct was co-injected with transposase RNA in the AB line at one-cell stage embryos and stable transgenic lines were subsequently established.

### Embryo medium (EM)

EM was made from 20XE2 (17.5 g NaCl, 0.75 g KCl, 4.9 g MgSO4-7H2O, 0.41 g KH2PO4, 0.12 g NaH2PO4-2H2O, into water for 1 L of total solution).

### Chemical treatments

A 250 mM VPA stock solution was made by dissolving sodium valproate salt (Sigma, P4543) into EM. A working solution of 250 µM VPA in EM was prepared from the stock solution. The EM was carefully maintained at pH 7.20. A stock solution of 12.5 mM D3T (Sigma, D5571) in DMSO was made. A working solution of 5 µM D3T was then made by dissolving the D3T stock solution in EM. The final concentration of DMSO in the working solution was 0.04%.

A new VPA stock and working solution was made and administered daily at 5pm, beginning at 6 hpf. Ten embryos/ per well were incubated in six-well plates; three wells received VPA treatments and three received only EM. Each well contained 7 ml of solution. Every day a new solution was made, and the old solution was replaced. The single dose of D3T was administered from 6 hpf to 18 hpf concurrently with 250 µM VPA, or as a pretreatment without VPA, then washed out and replaced with a 250 µM VPA only solution. Embryos were then treated daily with VPA as previously outlined.

### Confocal microscopy

Embryos and larvae were screened for expression of the fluorescent reporter, and three embryos per condition were embedded dorsal-side down in 1.5% low melting point agarose (LMA) (Gene Mate, E-3126-125) with 1:25 MESAB (Syndel, 200-226) in 10 mm glass-bottom microwells. Images were obtained on an Olympus Fluoview FV1000 confocal microscope using a 488 nm laser [*y304Et(cfos:Gal4); Tg(UAS:Kaede), y237Et(cfos:Gal4); Tg(UAS:Kaede)*] or a 546 nm laser [*Tg(NeuroD:tRFP)^w68^, photoconverted y237Et(cfos:Gal4); Tg(UAS:Kaede)*] with a 20X or 40X W lens. Images were collected at 640×640 pixels at 2μs/pixel. The HV of the 543 nm laser for experiments using the *Tg(NeuroD:tRFP)^w68^* line, was held constant across wild-type and treated embryos, so fluorescence could be compared between the two.

For time-lapses, multiple embryos (between three and five) were anesthetized and embedded dorsal-side down in 14 mm glass-bottom microwells. Valproic acid-treated embryos were embedded in 1.5% LMA,1:25 MESAB, and 250 µM VPA. Embedded control and VPA-treated embryos were covered with 3mL of the respective solution (EM or 250 µM VPA solution). Each embryo was imaged once every 10 min.

### Photoconversions

Following collection, *y237Et(cfos:Gal4); Tg(UAS:Kaede)* embryos were raised in the dark to prevent off-target photoconversion of Kaede. After larvae were prepared for imaging as previously described, photoconversion of Kaede was performed using a 405 nm laser at 1% power (8.6µW) for 5 s with a 40XW objective lens. Following dispersion of photoconverted Kaede (approximately 2 h), images were taken with a 40XW objective lens and a digital zoom of 4X at 800×800 pixels. All other settings for image capture remained the same as previously described.

Images were deconvolved using the deconvolution wizard in the Huygens Essential software and three dimensional skeletonized tracings were created using the SNT plugin (Arshadi et al., 2021) within Fiji (ImageJ). The investigator was blinded to the group allocation prior to creating skeletonized tracings. Measurement of neuronal characteristics such as tip number, terminal branch length, and convex hull size were derived directly from neuronal tracings through use of SNT's analysis tools. To measure the cell body position within the stratum periventriculare (SPV extension), neuronal tracings were overlayed with transmitted images to determine the neuropil boundary and the length of the primary extension prior to the neuropil boundary was measured using SNT's analysis tools. A similar process was used for measuring the length of MiP motor neuron projections within the larval spine.

### Enucleations

At 30 hpf embryos were dechorionated, anesthetized and embedded ventral-side down in 1.5% LMA. Using a sharpened tungsten needle, an incision was made in the epithelium anterior of the optic cup, and the optic cup and lens were gently pushed out. The embryos were un-embedded, returned to a dish with fresh EM, and grown to 5 dpf in standard conditions.

### Glutathione/glutathione disulfide redox potential analysis

At 6 hpf embryos were treated with DMSO (control vehicle) or D3T (5 µM). At 18 hpf, D3T was removed and VPA (250 µM) was added to half of the wells. At the indicated time points, 30 embryos were collected in 325 µL 5% perchloric acid and 0.2 M boric acid containing the internal standard ɣ-glutamylglutamate (10 µM) and stored at −20°C for 1/2 to 2 days. Glutathione and glutathione disulfide were measured with high performance liquid chromatography (HPLC) with fluorescence detection using ɣ-glutamylglutamate as an internal standard for each sample. Samples were derivatized to S-carboxymethyl, N-dansyl derivatives using the method described (Jones et al., 1998). GSH E_h_, GSH and GSSG concentrations were used in the Nernst equation E_h_=E_o_+(RT/nF)ln([GSSG]/[GSH]^2^).

### RT-qPCR

At 6 hpf embryos were treated with DMSO (control vehicle) or D3T (5 µM). At 18 hpf, 30 embryos were collected in 250 µl TRIzol reagent (Invitrogen) and stored at −20°C for 1/2-2 days. RNA was extracted using a Direct-zol RNA MiniPrep Kit (Zymo Research). cDNA was synthesized via reverse transcription using an iScript cDNA Synthesis Kit (Bio-Rad Laboratories) and used for quantitative real-time PCR (RT-qPCR) analysis using SYBR Green Detection Master Mixes (SABiosciences) on a StepOnePlus real-time PCR cycler (Applied Biosystems). All steps were performed per manufacturer's instructions. Specific forward and reverse primers for *Gclc, Hmox1a, Hmox1b, Nqo1, Gstp1, and* β*-actin* were synthesized by Integrated DNA Technologies (Table 1). β*-actin* was used as a housekeeping gene for all samples for normalization purposes. Relative levels of mRNA expression were calculated using the ΔΔC_T_ method relative to the expression of β*-actin* mRNA expression.

**Table 1.**
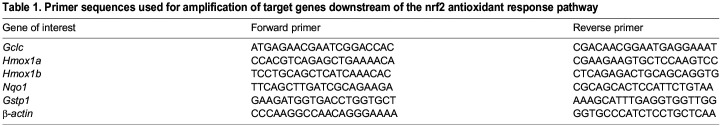
Primer sequences used for amplification of target genes downstream of the nrf2 antioxidant response pathway

### OT Quantifications

Images were quantified using Fiji (ImageJ). To obtain length and width, a rotated rectangle was placed over each lobe of the OT from the top innermost corner to the bottom outermost corner. The width of the rectangle was adjusted so that the entire lobe fit within the rectangle. The length and width of the rectangle was then measured. To obtain the depth, the z-stack image was resliced, and a rotated rectangle was placed in a similar manner, this time only measuring its width providing the depth of the OT. Each measurement was repeated for the other half of the OT, and lobe measurements were averaged to provide the average lobe length, width, and depth per embryo or larvae.

For neuropil volume quantifications, the neuropil was traced in each z-stack slice using Fiji (ImageJ). Tracings were performed on each OT lobe separately and at a 200% zoom to ensure accuracy. The neuropil area measurements for each z-stack slice were added to obtain an overall neuropil area and this was multiplied by the step size (2.2 µm) for an estimated volume. This procedure was repeated for the other lobe and the two results were averaged to provide the final neuropil volume per lobe. Quantification of OT tRFP expression followed a similar workflow, except images were thresholded prior to tracing. After obtaining the tRFP volume, results were converted to fold change compared to control within that timepoint. Microscope settings were kept consistent within the same day allowing for comparison.

## Supplementary Material

10.1242/biolopen.059567_sup1 Supplementary information
