# Supplementary material for: Valproic acid affects neurogenesis during early optic tectum development in zebrafish
Source: Biol Open. 2023 Jan 31;12(1):bio059567. doi: 10.1242/bio.059567 (PMC9916031; doi:10.1242/bio.059567)
Supplement: Supplementary information [file biolopen-12-059567-s1.pdf]

Supplementary Figure 1

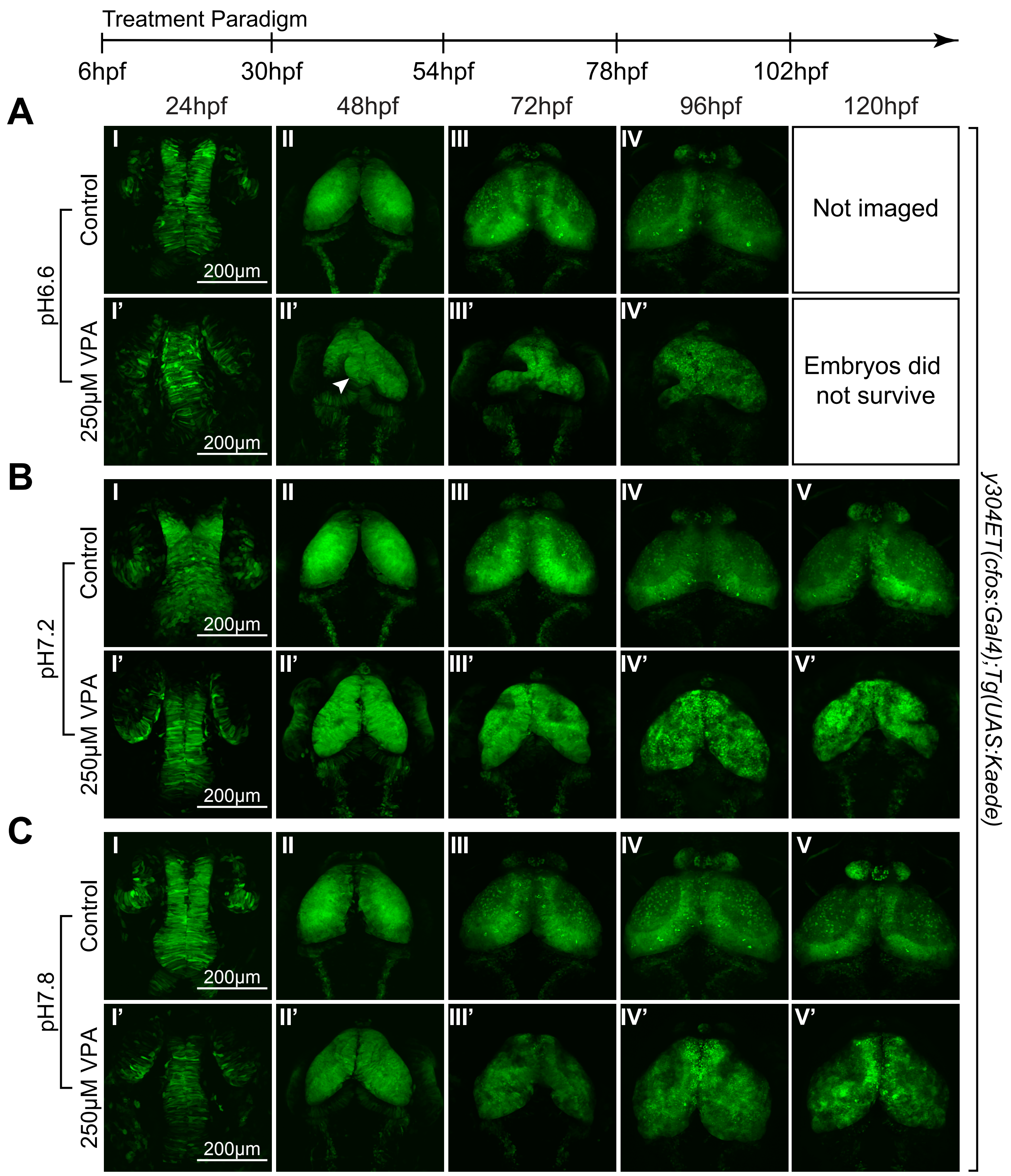

Fig. S1. OT imaging reveals the ability of pH to modulate the effects of VPA in the OT. Daily images of control (I-V) and treated (I'-V') *y304Et(cfos:Gal4);Tg(UAS:Kaede)* embryos with 250μM VPA dissolved in embryo media pH6.6 (A), pH7.2 (B), and pH7.8 (C). Treated embryos were continuously exposed to VPA solution from 6-120hpf. (A) VPA-treated embryos incubated at pH6.6 (I'-IV') exhibited strong phenotypes such as twisted tecti as soon as 48hpf (arrow, II') and no neuropil formation at 96hpf (IV'). Moreover, they did not survive for imaging at 120hpf. (B) VPA-treated embryos incubated at pH7.2 (I'-V') did not exhibit twisting of the tecti as seen at pH6.8 (A), but they displayed a smaller OT and lacked neuropil formation at 72-120hpf (III'-V'). (C) VPA-treated embryos incubated at pH7.8 (I'-V') presented a slightly weaker phenotype,characterized by an increase in OT size at 120hpf (V') compared to embryos incubated at pH7.2 (B) or pH6.8 (A). *Note: OT development in control embryos was unaffected by pH.*

Biology Open • Supplementary information

Supplementary Figure 2

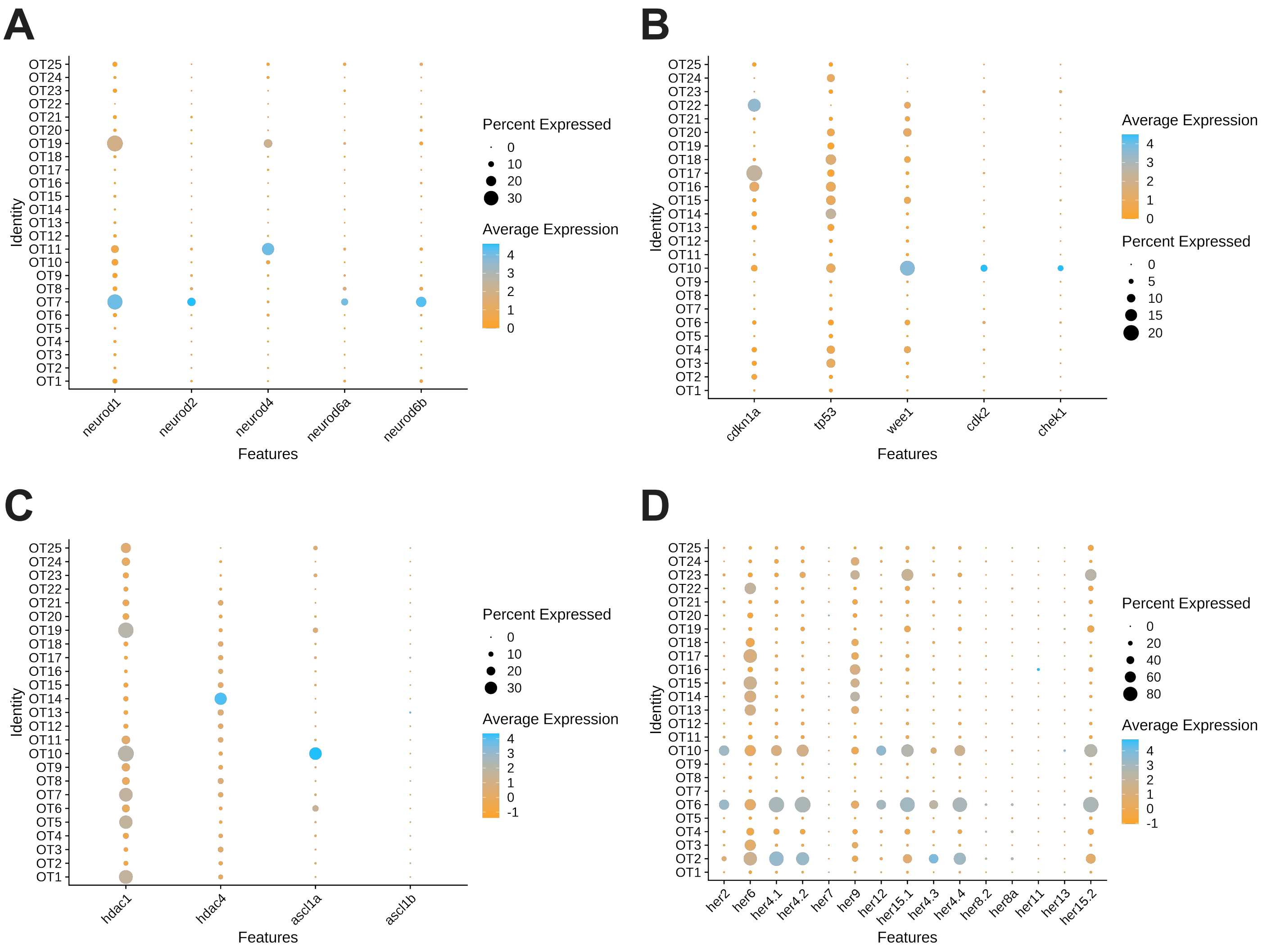

Fig. S2. Presence of genes of interest in the OT at 7dpf determined by scRNA-seq data. Relative expression of various genes of interest within cellular subtypes of the OT. Point size indicates percentage of cells expressing the gene within a population. Point color indicates average gene expression within a population. (A) At 7dpf all *NeuroD* genes were expressed in the OT with *NeuroD1* found in the greatest number of OT clusters. Note that although *NeuroD1* can be found in many clusters, it is largely sequestered to OT7 and OT19 by 7dpf. (B) Expres-sion of various cell cycle genes reveal that *cdkn1a* and *tp5d* are found throughout the OT at 7dpf. In contrast, *wee1*, *cdk2*, and *chek1*, are largely found in OT10, a cluster identified as developing neuronal 39. (C) Expression profiles show that both *HDAC1* and *HDAC4* are present in the OT at 7dpf. *Ascl1a*, an important downstream target of the Notch pathway, is also found in the OT at 7dpf, although *ascl1b* does not appear to be present. (D) Various *HER* genes, additional downstream targets of the Notch pathway, show widespread expression between clusters and a high percentage of cells expressing within those clusters at 7dpf.

Biology Open • Supplementary information

Supplementary Figure 3

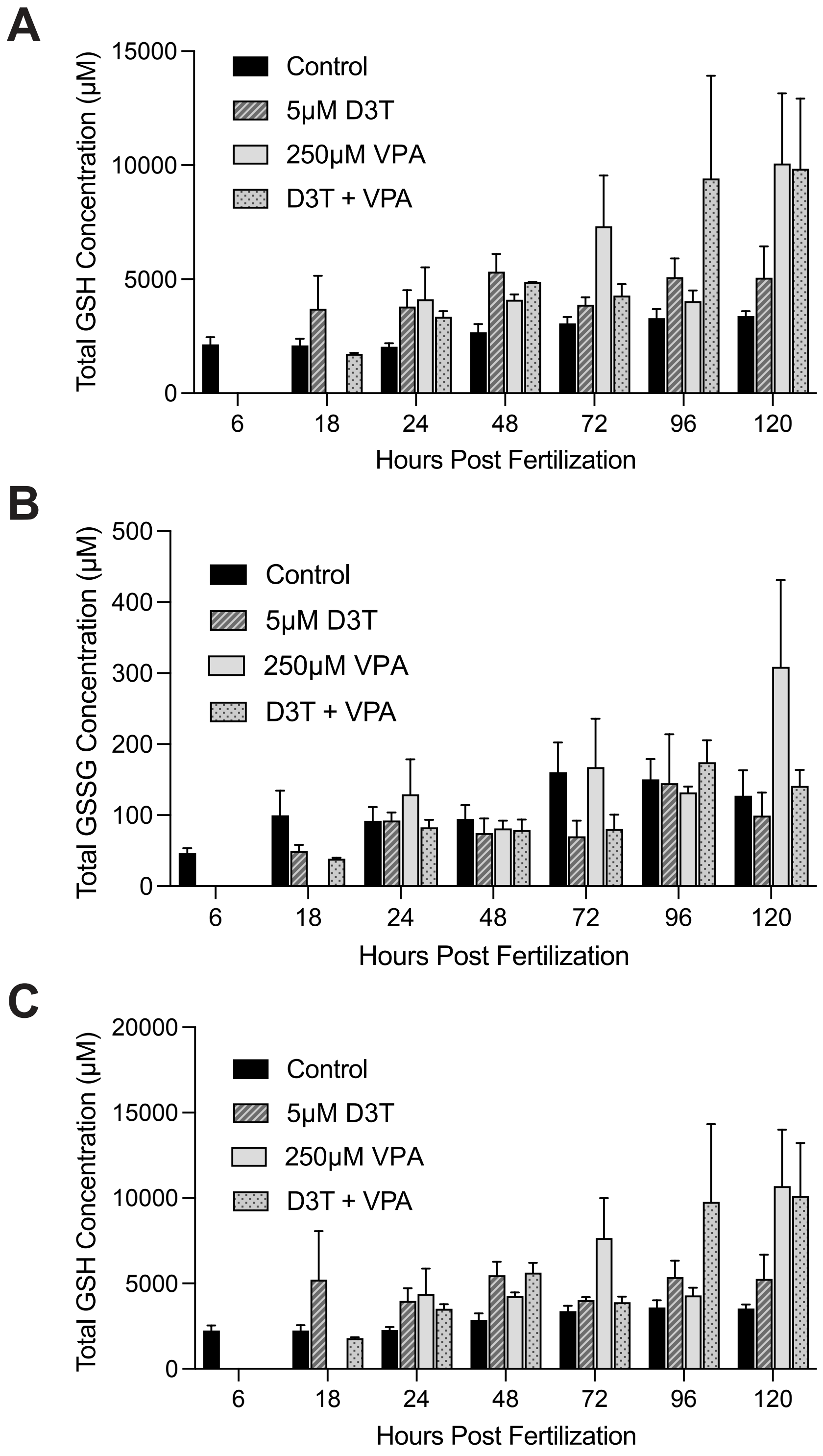

Fig. S3. GSH(reduced glutathione), GSSG(oxidized glutathione), and GSHTot (reduced and oxidized) during development. GSH (A), GSSG(B), and GSHTot (C) concentrations over the course of embryonic development (6-120hpf) for control, 5µM D3T, 250µM VPA,and 5µM D3T+ 250µM VPA-treated embryos. D3T+VPA-treated embryos received a 12-hour D3T pretreat-ment from 6-18hpf, following which D3T+VPA and VPA-treated embryos were continuously exposed to VPA from 18-120hpf. Concentration was determined by protein quantification assay and HPLC. By 5dpf all treated groups showed increased GSH(A) and GSHTot(C) compared to controls. All data points represent n=3 pools of 30 embryos each. Data plotted as mean ±SEM.

Biology Open • Supplementary information

Supplementary Figure 4

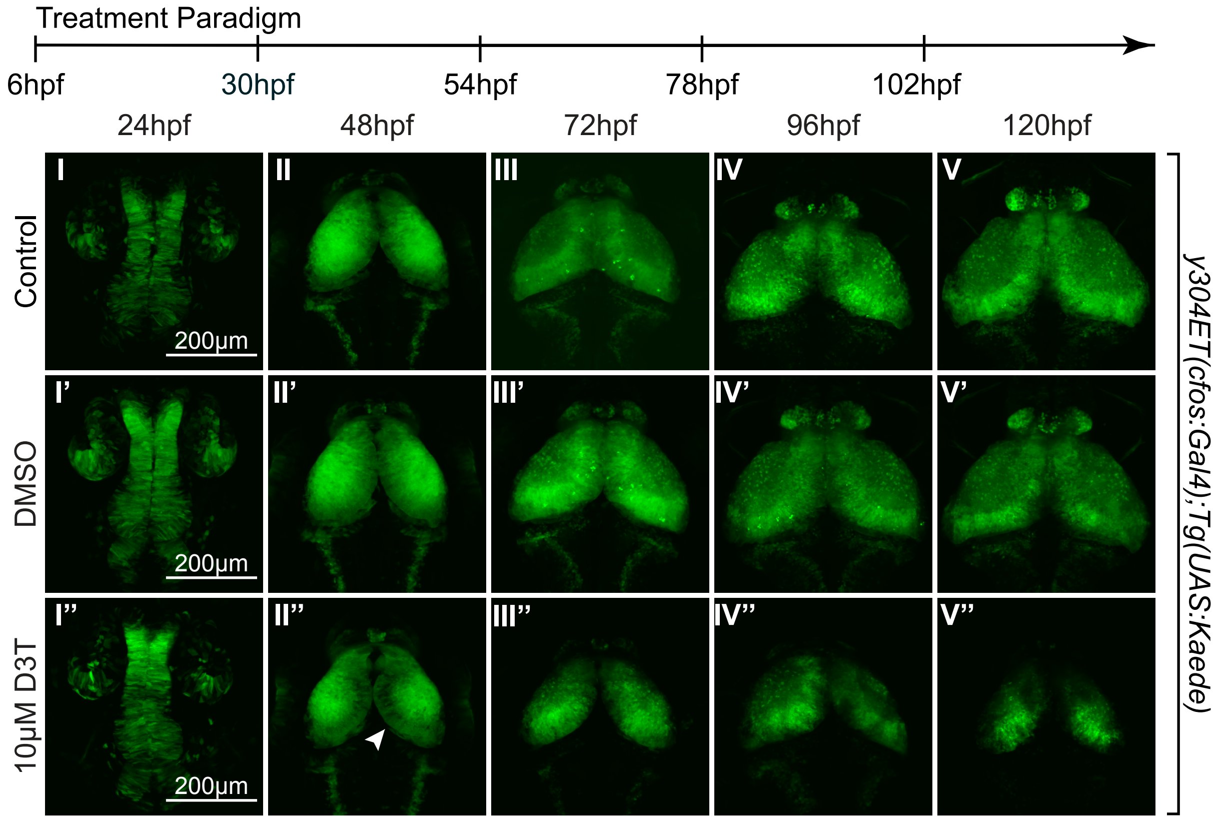

Fig. S4. Extended dosing paradigm demonstrates that prolonged exposure to high concentrations of D3T alters OT development. Daily images of control (I-V), DMSO (I'-V'), and 10µM D3T-treated (I''-V'') *y304Et(cfos:Gal4); Tg(UAS:Kaede)* embryos. Treated embryos were continuously exposed to DMSO (I'-V') or 10µM D3T (I''-V'') from 6-120hpf. At 48hpf embryos treated with 10µM D3T (II'') showed increased columnar neuroepithelial cells (arrow, II''), and an overall decrease in size, when compared to both control (II) and DMSO embryos (II'). This delay became increasingly more apparent with D3T-treated larvae showing severe malformation in the OT at 120hpf (V''). DMSO-treated embryos (I'-V') did not exhibit noticeable deviations from control embryos (I-V) at any measured timepoints.

Biology Open • Supplementary information

Supplementary Figure 5

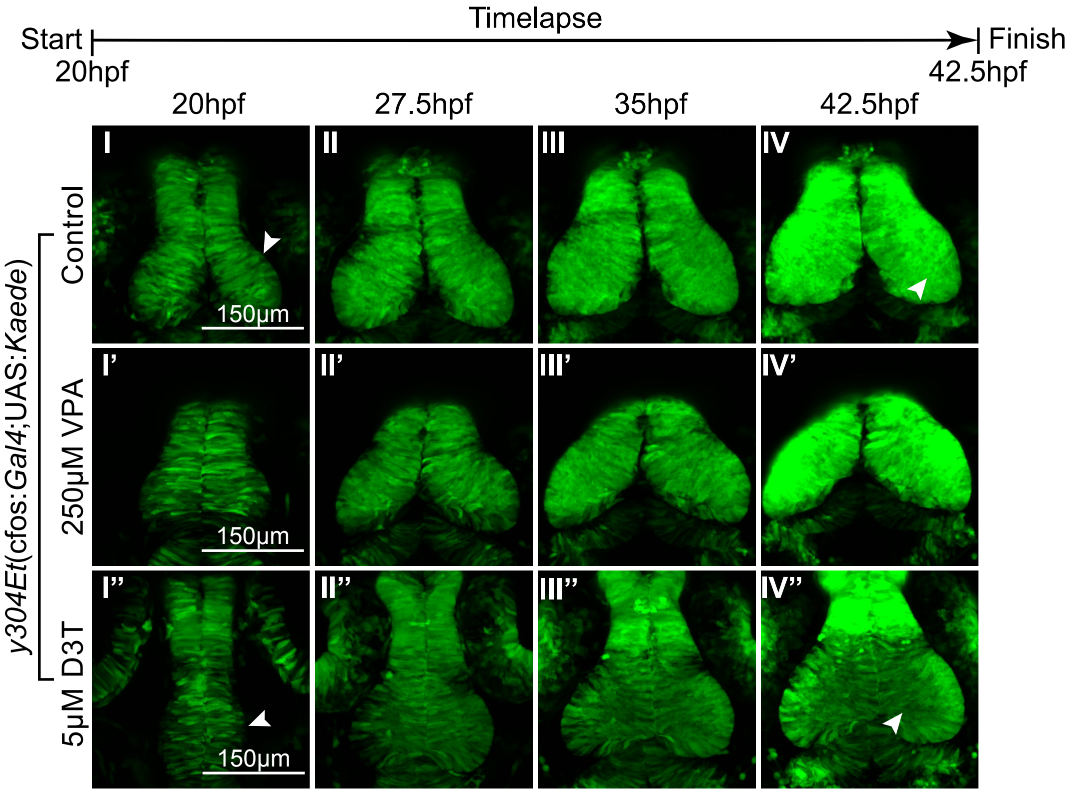

Fig. S5. Timelapse immediately after D3T pretreatment indicates that D3T does not ameliorate the effects of VPA on OT development during the critical period. Images captured at regular intervals from a 20-42.5hpf timelapse of *y304Et(cfos:Gal4);Tg(UAS:Kaede)* control (I-IV), 250µM VPA (I’-IV’) and 5µM D3T+250µM VPA-treated (I’’-IV’’) embryos. D3T+VPA-treated embryos received a 12-hour D3T pretreatment from 6-18hpf following which D3T+VPA and VPA embryos were continuously exposed to VPA from 18-42.5hpf. Pretreatment with 5µM D3T (I’’-IV’’) failed to remedy the effects of VPA on OT development when compared with control (I-IV), and VPA-treated (I’-IV’) embryos. At 20hpf D3T+VPA embryos (arrow, I’’) lack proliferation of the posterior neuroepithelium seen in control embryos (arrow, I). By 42.5hpf D3T+VPA embryos exhibit persistent columnar neuroepithelial cells (arrow, IV’’) in contrast to control embryos which display more rounded cells indica-tive of differentiation (arrow, IV).

Biology Open • Supplementary information

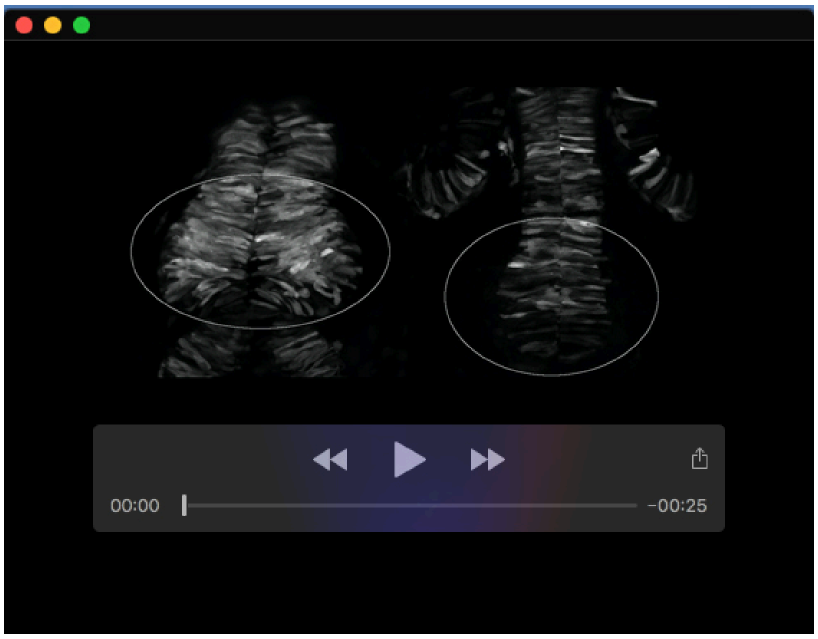

Movie 1. *y304Et(cfos:Gal4); Tg(UAS:Kaede)* Timelapse from 22.5hpf-30hpf, control and 250μM VPA treated

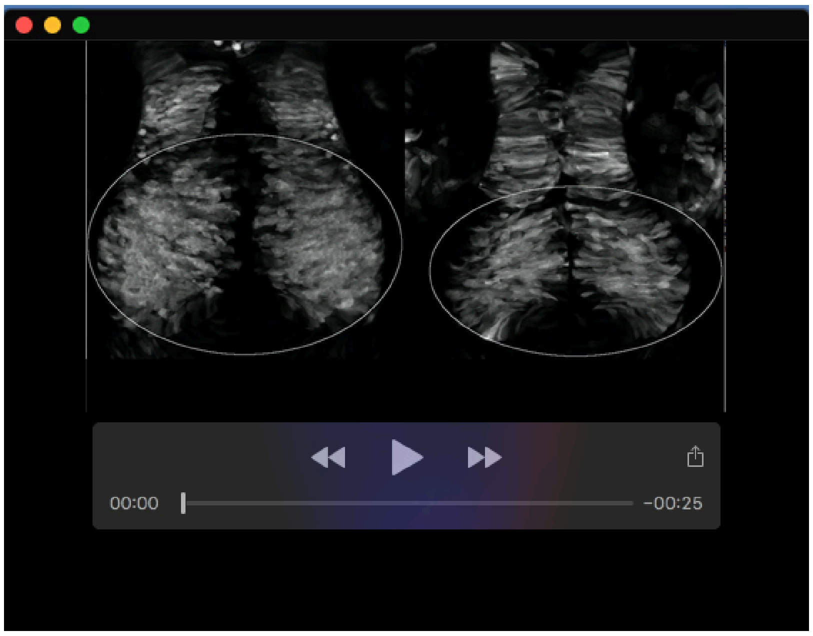

Movie 2. *y304Et(cfos:Gal4); Tg(UAS:Kaede)* Timelapse from 30hpf-43.5hpf, control 250μM VPA treated.

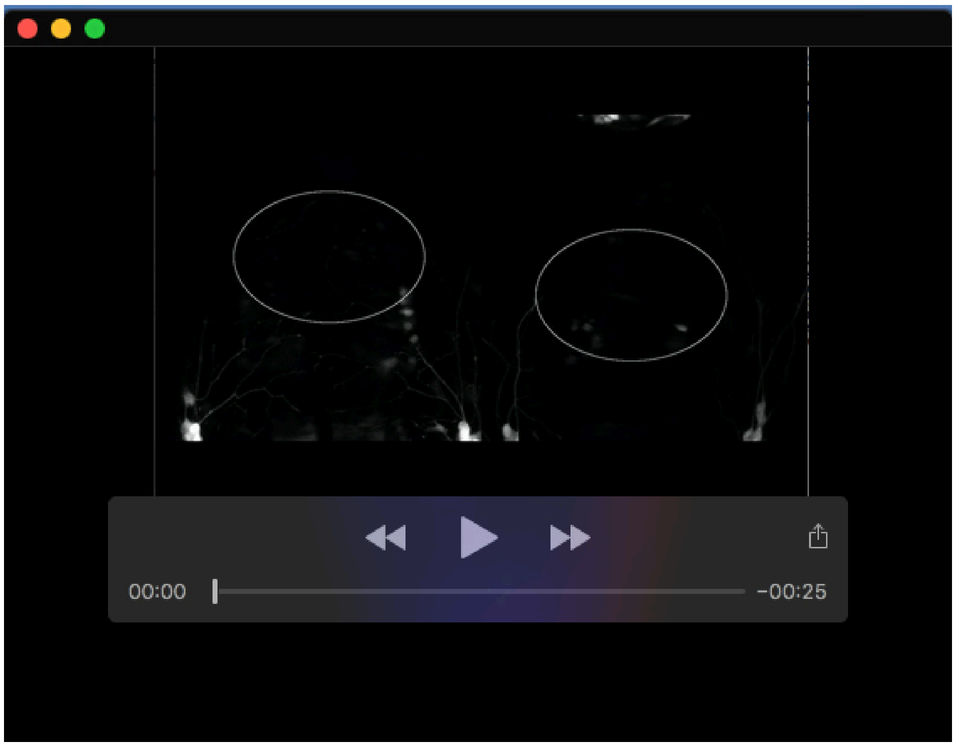

Movie 3. *Tg(NeuroD:tRFP)^{w68}* Timelapse from 22.5hpf-30hpf, control and 250μM VPA treated

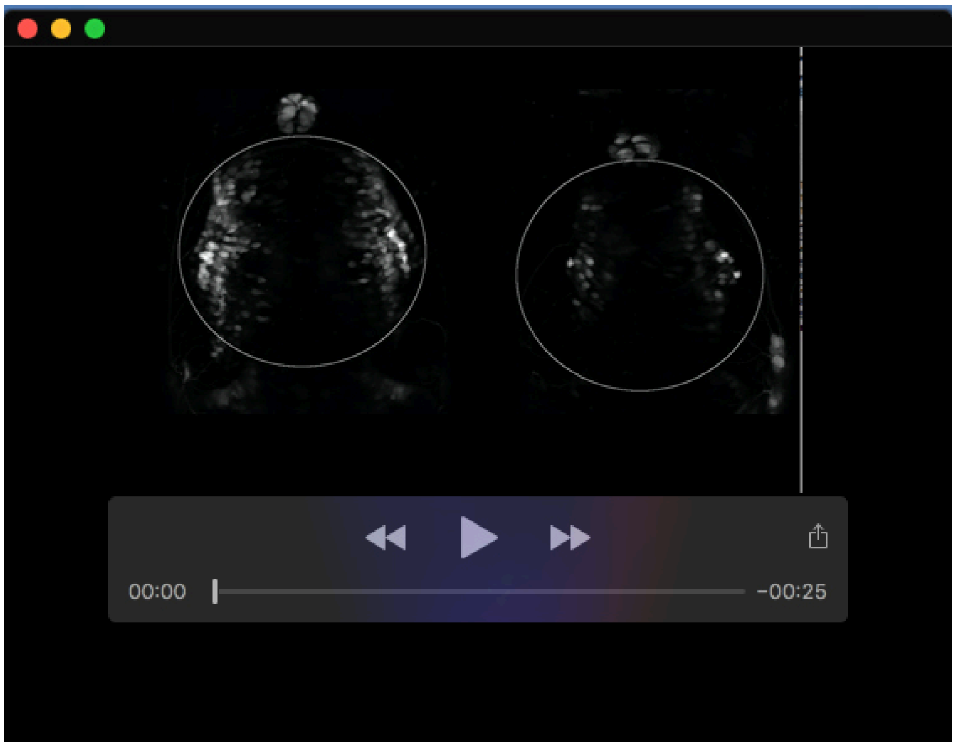

Movie 4. *Tg(NeuroD:tRFP)^{w68}* Timelapse from 30hpf-43.5hpf, control and 250μM VPA treated

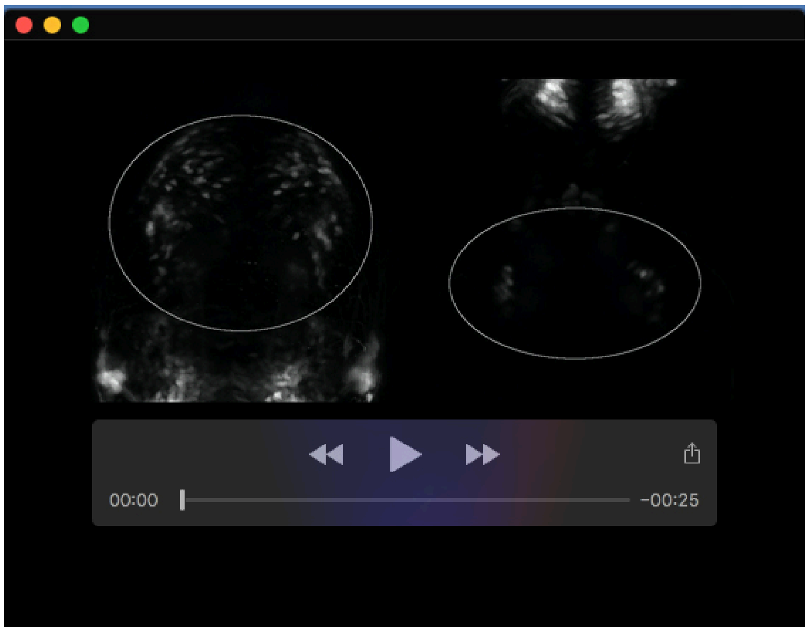

Movie 5. *Tg(NeuroD:tRFP)*^{w68} Timelapse from 30hpf-43.5hpf, control and 250µM VPA treated; different embbeding angle.

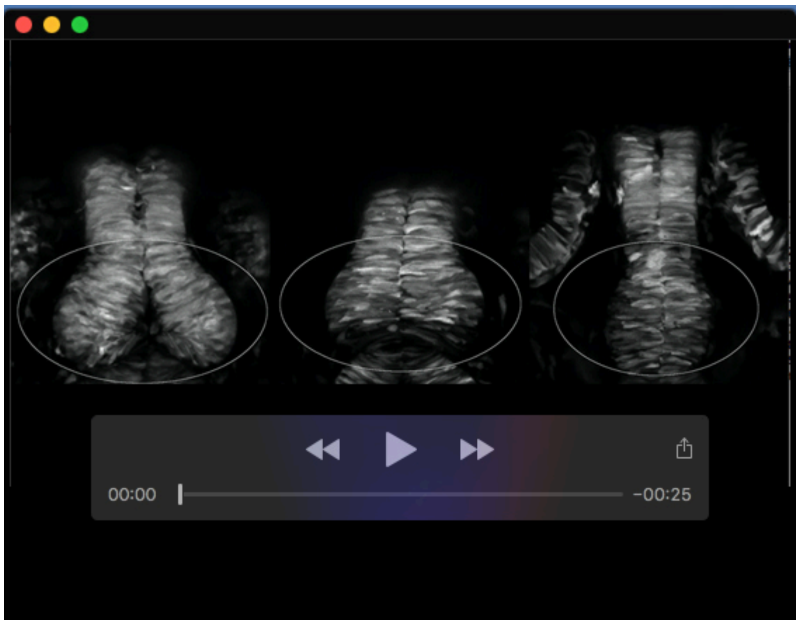

Movie 6. *y304Et(cfos:Gal4); Tg(UAS:Kaede)* Timelapse from 20hpf-42.5hpf, control, 250µM VPA, 5µM D3T.

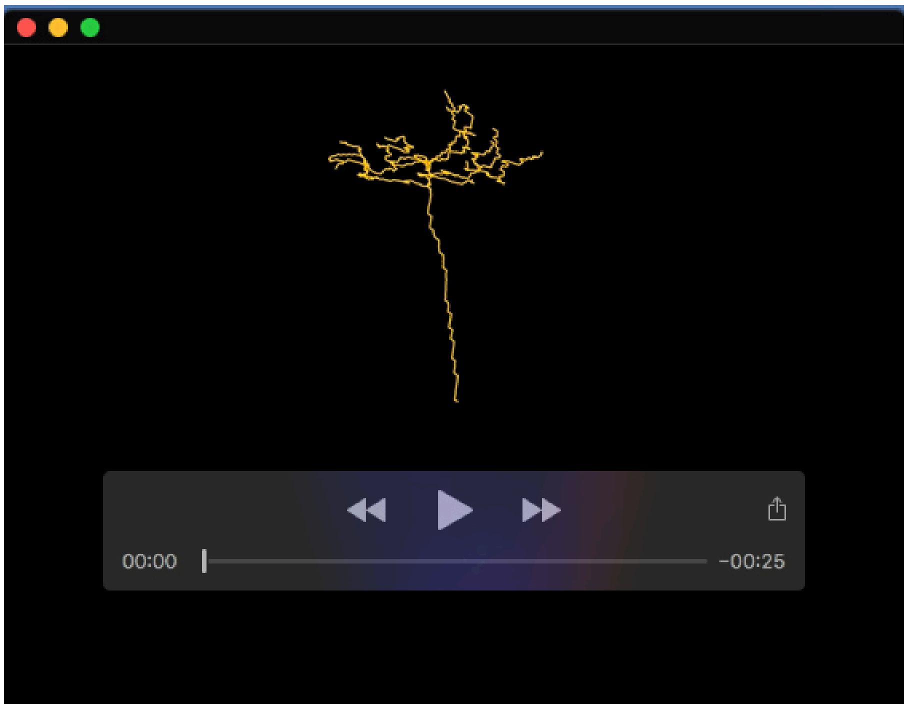

Movie 7. Single PVIN photoconverted neuron subtype 1

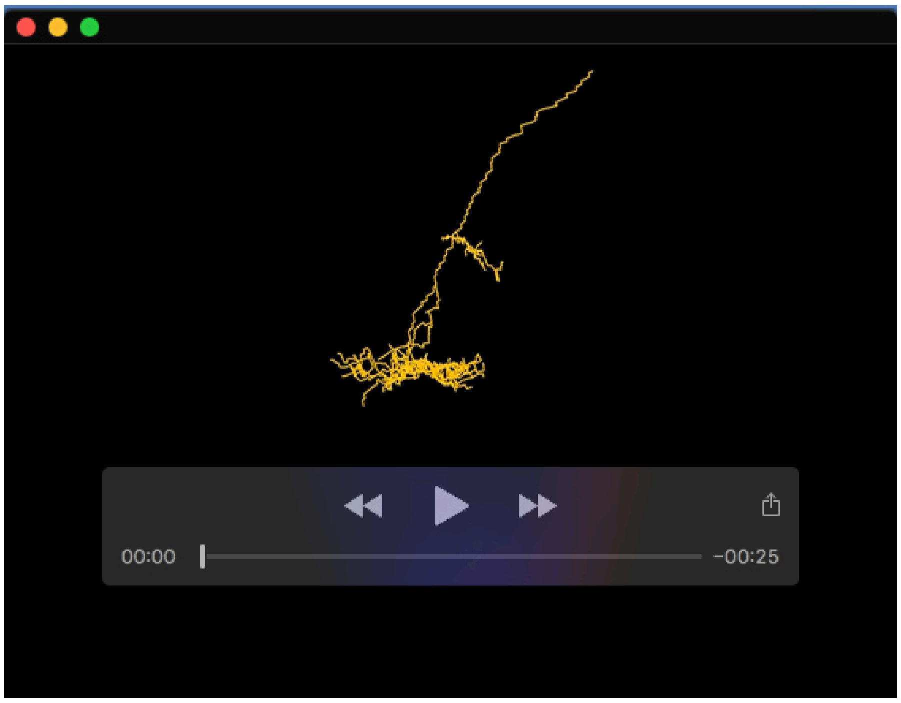

Movie 8. Single PVIN photoconverted neuron subtype 2

Biology Open • Supplementary information

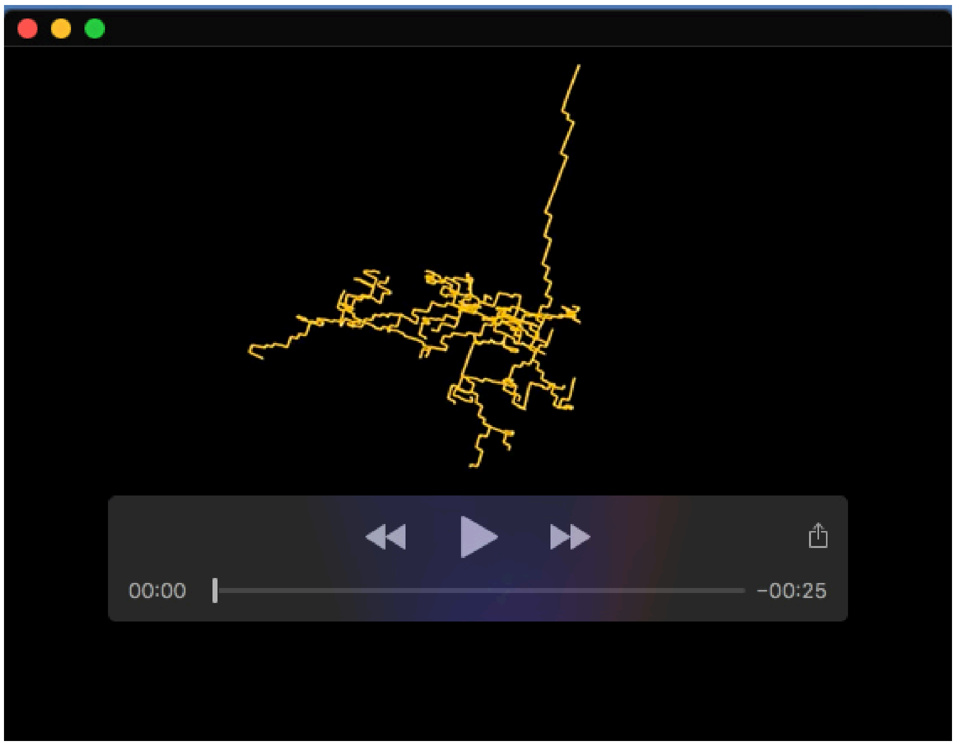

Movie 9. Single PVIN photoconverted neuron subtype 3 Supplementary

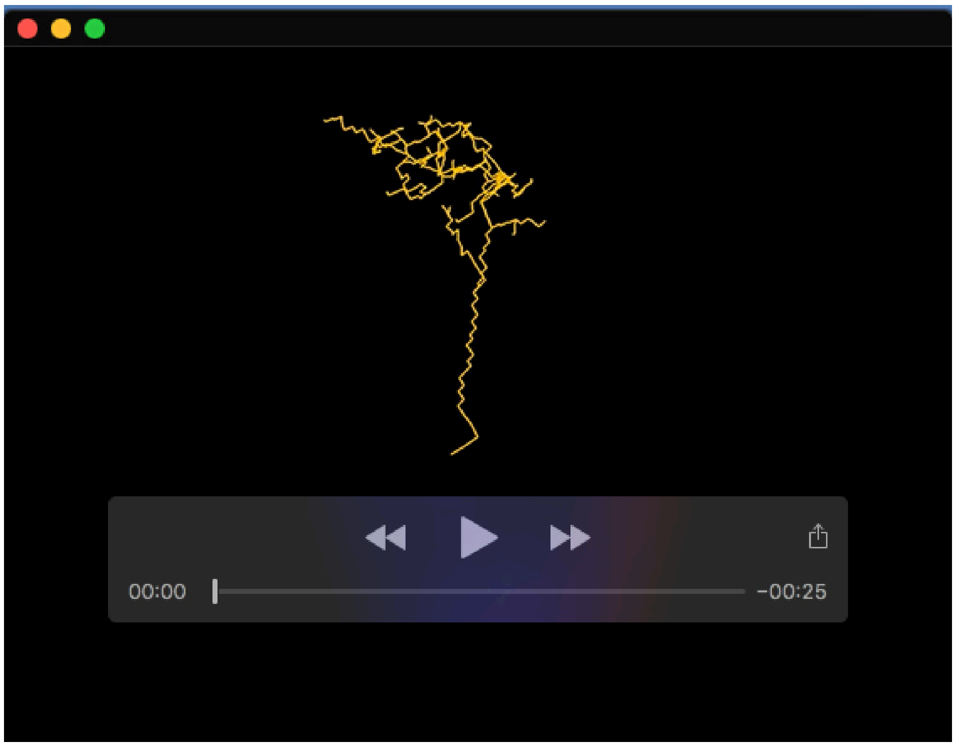

Movie 10. Single PVIN photoconverted neuron subtype 4

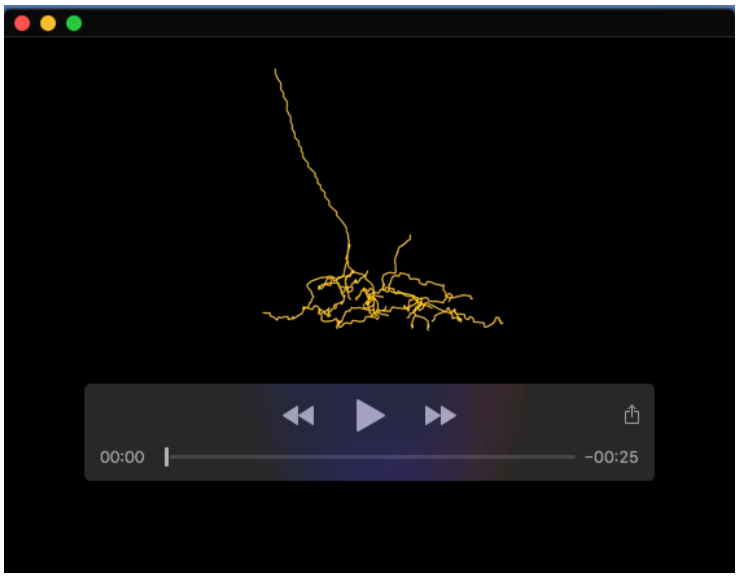

Movie 11. Single PVIN photoconverted neuron subtype 5
